# CMTM4 regulates angiogenesis by promoting cell surface recycling of VE-cadherin to endothelial adherens junctions

**DOI:** 10.1007/s10456-018-9638-1

**Published:** 2018-08-10

**Authors:** Ihsan Chrifi, Laura Louzao-Martinez, Maarten M. Brandt, Christian G. M. van Dijk, Petra E. Bürgisser, Changbin Zhu, Johan M. Kros, Marianne C. Verhaar, Dirk J. Duncker, Caroline Cheng

**Affiliations:** 1 Experimental Cardiology, Department of Cardiology, Thoraxcenter, Erasmus University Medical Center, Rotterdam, The Netherlands; 2 Division of Internal Medicine and Dermatology, Department of Nephrology and Hypertension, University Medical Center Utrecht, Utrecht, The Netherlands; 3 Netherlands Heart Institute, Utrecht, The Netherlands; 4 Department of Pathology, Erasmus University Medical Center, Rotterdam, The Netherlands

**Keywords:** Endothelial cells, Angiogenesis, Adherens junctions, VE-cadherin, CMTM4

## Abstract

Vascular endothelial (VE) cadherin is a key component of endothelial adherens junctions (AJs) and plays an important role in maintaining vascular integrity. Endocytosis of VE-cadherin regulates junctional strength and a decrease of surface VE-cadherin reduces vascular stability. However, disruption of AJs is also a requirement for vascular sprouting. Identifying novel regulators of endothelial endocytosis could enhance our understanding of angiogenesis. Here, we evaluated the angiogenic potential of (CKLF-like MARVEL transmembrane domain 4) CMTM4 and assessed in which molecular pathway CMTM4 is involved during angiogenesis. Using a 3D vascular assay composed of GFP-labeled HUVECs and dsRED-labeled pericytes, we demonstrated in vitro that siRNA-mediated CMTM4 silencing impairs vascular sprouting. In vivo, CMTM4 silencing by morpholino injection in zebrafish larvae inhibits intersomitic vessel growth. Intracellular staining revealed that CMTM4 colocalizes with Rab4^+^ and Rab7^+^ vesicles, both markers of the endocytic trafficking pathway. CMTM4 colocalizes with both membrane-bound and internalized VE-cadherin. Adenovirus-mediated CMTM4 overexpression enhances the endothelial endocytic pathway, in particular the rapid recycling pathway, shown by an increase in early endosomal antigen-1 positive (EEA1^+^), Rab4^+^, Rab11^+^ , and Rab7^+^ vesicles. CMTM4 overexpression enhances membrane-bound VE-cadherin internalization, whereas CMTM4 knockdown decreases internalization of VE-cadherin. CMTM4 overexpression promotes endothelial barrier function, shown by an increase in recovery of transendothelial electrical resistance (TEER) after thrombin stimulation. We have identified in this study a novel regulatory function for CMTM4 in angiogenesis. CMTM4 plays an important role in the turnover of membrane-bound VE-cadherin at AJs, mediating endothelial barrier function and controlling vascular sprouting.

**Electronic supplementary material:**

The online version of this article (10.1007/s10456-018-9638-1) contains supplementary material, which is available to authorized users.

## Introduction

Angiogenesis is a critical process that occurs during embryonic development, is re-activated in wound healing, and is often disturbed in disease. Abnormalities in new vessel formation, represented by either excessive or insufficient growth, contribute to a wide range of disorders, including cancer and cardiovascular diseases [1].

Acquiring and maintaining vascular homeostasis is crucial for the formation and stabilization of new blood vessels during angiogenesis. In order for the endothelial cells (ECs), that form the inner lining of new blood vessels, to function in an integrated manner, specific junctions consisting of cell adhesion molecules are required. From these junctions, adherens junctions (AJs) are critical for cell–cell adhesion and are tightly regulated by complex molecular pathways. Vascular endothelial (VE) cadherin is the main cell adhesion molecule found in endothelial AJs and plays an important role in maintaining endothelial barrier function [2, 3]. Blocking VE-cadherin by the use of antibodies has a negative effect on endothelial survival and increases vascular permeability [4]. In addition, VE-cadherin-deficient mice suffer from severe vascular defects, including impaired vascular sprouting, limited organization of ECs into large mature vessels, and vessel regression, resulting in death at mid-gestation [5, 6].

AJs are dynamic structures from which the amount of membrane-bound VE-cadherin that can undergo homophilic interactions with neighboring cells can be actively regulated, thereby modulating the junctional adhesive strength. A reduction in membrane-bound VE-cadherin will result in microvascular destabilization and loss of barrier function. However, a decrease in adhesive strength is required for the disruption of AJs in order to allow EC migration and morphological adaptation into tip and stalk cells during vascular sprouting.

Endocytosis is a form of active transport that is commonly used by cells to take up large secreted molecules or membrane-bound proteins, such as VE-cadherin, into the cytosol for processing. VE-cadherin is taken up in specialized regions called clathrin-coated pits that bud from the membrane to form clathrin-coated vesicles. These vesicles then fuse with early endosomes in which the endocytosed cargo is sorted for recycling, lysosomal, or other trafficking routes [7]. Each distinct trafficking step is controlled by a subset of Rab GTPases, making these enzymes ideal markers to monitor the transport route of endocytosed proteins [8]. Rapid endocytosis of VE-cadherin and the subsequent disassembly of AJs is promoted by vascular endothelial growth factor A (VEGFA), making the ligand for the VEGF Receptor 1 and 2 (VEGFR1 and 2) an active regulator of vascular permeability [9].

Whereas endocytosis plays a vital role in modulating vascular permeability by controlling the adhesive strength in AJs, little is known about important regulators involved in the early endocytic and subsequent trafficking routes in ECs. Moreover, the exact contribution of endocytosis regulators to the angiogenic process remains to be further elucidated. To identify novel regulators of angiogenesis, we performed a transcriptome analysis of mouse embryos, comparing VEGFR2^+^ endothelial progenitor cells with the VEGFR2-negative cell population. We identified an enriched expression of several members of the CKLF-like Marvel Transmembrane Domain-containing gene (CMTM) family in the VEGFR2^+^ population. In humans, this family comprises 9 genes (CKLF and CMTM1-8) that all contain a MAL (myosin and lymphocyte) And Related proteins for Vesicle trafficking and membrane Link (MARVEL) domain [10]. Several studies on CMTM members reveal that they can prevent growth and invasion of different cancer types [11–18]. In a recent study, we revealed that CMTM3 has a specialized function in ECs, where it is involved in regulating the early endocytosis of VE-cadherin during vascular growth [19]. However, the putative function of other CMTM members in endothelial endocytosis, regulation of vascular permeability, and angiogenesis remains to be elucidated. Increasing our understanding of other regulators of angiogenesis is highly important for the development of novel therapeutic targets.

In this study, we investigated the angiogenic potential of CMTM4 and assessed in which molecular pathways CMTM4 is involved in ECs. To our knowledge, CMTM4 is until now only known for its potential to act as a tumor suppressor gene [14]. Our data demonstrated that CMTM4 regulates angiogenesis by promoting cell surface recycling of VE-cadherin to endothelial AJs.

## Materials and methods

### Cell culture

Primary human umbilical vein endothelial cells (HUVECs) were purchased from Lonza and maintained in EGM2 medium (Lonza) with 100 Uml^−1^ penicillin-streptomycin (PS). Primary human brain-derived vascular pericytes were purchased from ScienCell and maintained in DMEM + 10% FCS with 100 Uml^−1^ PS. At passage 1, HUVECs were transduced with lentiviral GFP construct (HUVECs-GFP) and pericytes with lentiviral dsRED construct (pericytes-dsRED) and selected for puromycin (2.5 ug/ml) resistance as previously described [19]. All cells were used between passages 3 and 6 and cultured at 37 °C in a humidified atmosphere containing 5% CO_2_.

### qPCR analysis

Total RNA was isolated from cultures (single-cell monolayers and co-cultured HUVECs and pericytes) using RNeasy kit (Qiagen) according to the manufacturer’s instructions. The purity and concentrations of RNA were quantified by using Nanodrop (ND-1000) absorbance measurements at 260/280 nm and/or capillary electrophoresis (Agilent 2100 Bioanalyser). The iScript synthesis kit (Biorad) was used for cDNA synthesis according to the manufacturer’s instructions. Gene expression was determined using quantitative real-time PCR (qPCR) according to the SYBR-Green-Cycler IQ5 detection protocol (Biorad). The primer sequences are listed in Supplementary Table 1. All results were normalized for the expression of house-keeping gene β-actin.

### Short interference RNA (siRNA)

CMTM4 or CMTM3 knockdown in HUVECs and HUVECs-GFP was achieved by using either a mix of 4 complementary siRNA sequences or 2 sets of individual siRNA sequences directed against the mRNA of CMTM4 or CMTM3 (ThermoScientific). As a negative control, cells were either untreated or transfected with a mix of 4 non-targeting siRNA sequences. The sequences are listed in Supplementary Table 2.

### Adenoviral transduction

Human recombinant CMTM4 or CMTM3 adenovirus were produced using the Gateway pAd/CMV/V5-DEST vector kit and ViraPower™ Adenoviral Expression System kit, according to the manufacturer’s instructions (Invitrogen). Fully sequenced Human CMTM4 or CMTM3 cDNA were obtained from OriGene and Lifesciences, respectively, cloned in a pCMV-SPORT6 vector. To amplify the CMTM4 gene from the obtained clone and simultaneously convert it with *att*B sites, a PCR was conducted with full-length *att*B-CMTM4-specific primers: forward oligo’s 5′- GGGGACAAGTTTGTACAAAAAAGCAGGCTTCACCATGCGGAGCGGCGAGGAGCTGGACGG-3′ and reverse oligo’s 5′- GGGGACCACTTTGTACAAGAAAGCTGGGTTTCACGTGTCCAGGCGCTGGATCTCAGG-3′. The full-length *att*B-CMTM4 amplicons was then cloned into a pDONR™ 221 vector to create an entry clone, according to BP Clonase™ II protocol. Full-length cDNAs of CMTM3 with attB-site was amplified by PCR using specific forward oligos 5′- GGGGACAAGTTTGTACAAAAAAGCAGGCTTCACCATGTGGCCCCCAGACCCCGACC-3′ and reverse oligo’s 5′- GGGGACCACTTTGTACAAGAAAGCTGGGTTTCAGTCAGAGTCCGAGTCGGAATTCTC-3′. Moreover a HIS tag was placed at the *C*-terminal region of CMTM3 (CMTM3-HA) using the RV primer 5′- GGGGACCACTTTGTACAAGAAAGCT GGGTGGTCAGAGTCCGAGTCGGAATTCTC-3′. Amplicons of CMTM3 and CMTM3-HA were cloned into a pDONRTM 221 vector via BP ClonaseTM II enzyme mix to create an entry clone. DNA sequencing validated the obtained CMTM4 or CMTM3 sequence. Using LR-reaction II (Invitrogen), the CMTM4 or CMTM3 reading frame cassettes were cloned into pAd/CMV/V5-DEST (Invitrogen) expression vectors according to the manufacturer’s protocol. After verification by DNA sequencing, the pAd/CMV/V5-DEST plasmids were linearized by *Pac*1 restriction and transfected with Lipofectamine 2000 (Invitrogen) into 293A cells. When 80% of the infected cells formed crude viral vesicles, the cells were harvested, followed by three cycles of freeze/thawing to get crude viral lysate (CVL), and the virus was purified using cesium chloride gradient.

### Subcellular fractioning and protein extraction

To separate cytoplasmic proteins from the membrane-bound proteins, i.e., the soluble and insoluble fraction, respectively, HUVECs received a PBS wash twice and were harvested in lysis buffer [140 mM NaCl, 10 mM Tris (pH 7.6), 1 mM EDTA, 1% Triton X-100, 0.05% SDS, 1X protease inhibitor cocktail, 1 mM PMSF] 48-h post-transfection. The lysates were incubated on ice for 20 min and were centrifuged at 13,000 g for 12 min to separate the soluble from the insoluble fraction. The pellet was then solubilized in lysis buffer with 2% SDS. Both fractions were subjected to SDS–PAGE and immunoblotting.

### Western blot

Protein concentration was determined by the Bradford method (Pierce BCA Protein Assay kit). Equal amounts of sample were loaded on a sodium dodecyl sulfate–polyacrylamide gel electrophoresis (SDS–PAGE) and transferred onto a nitrocellulose membrane (Pierce) at 4 °C overnight. Membranes were blocked and probed with primary antibodies according to the manufacturer’s instructions (Supplementary Table 3). Protein bands were visualized with Li-Cor secondary antibodies and detection system (Westburg) according to the manufacturer’s instructions. The detected protein expressions were normalized with β-actin.

### Collagen-based 3D co-culture

HUVEC-GFP and pericyte-dsRed were co-cultured at a 5:1 ratio in a 3D bovine collagen type 1 (2 mg/ml) environment (Gibco), supplemented with EBM-2 medium (Lonza), ascorbic acid, fibroblast growth factor, 2% FCS, SCF-1, SDF-1α, and IL-3 (i.e., co-culture medium), as previously described [20]. Images were taken 2 days after seeding to assess the sprouting capacities of ECs and at day 5, when lumenized micro-capillary structures are formed. Images were taken with fluorescence microscopy with a 2x objective. Images were analyzed based on the number of junctions, the number of tubules, and the tubule length using AngioSys 2.0 software.

### VE-cadherin internalization assay and immunofluorescence

The internalization assay was performed at 48-h post-transfection as previously described [9]. Briefly, HUVECs were grown on coverslips to confluence before labeling the cell surface-exposed VE-cadherin by incubation with mouse anti-human VE-cadherin (Supplementary Table 3) at 4 °C for 1 h. Movement of labeled VE-cadherin was monitored by placing the cells at 37 °C for 1 h, in basal medium with or without VEGF (50 ng/ml; Preprotech), after which the cells were either washed with PBS (PBS supplemented with 1.8 mM CaCl2 and 1 mM MgCl2) or with a mildly acidic buffer [2 mM PBS-glycine (pH 2.0), 15 min] to remove membrane-bound antibodies and reveal the internalized labeled VE-cadherin. Cells were fixed with 4% paraformaldehyde for 30 min, followed by a blocking and permeabilization step for 30 min in PBS with 1% BSA and 0.5% Triton-X. Secondary antibody (Supplementary Table 3) incubation was performed for 1 h at room temperature, followed by a 30-min incubation step to label cytoskeletal F-actin with rhodamine phalloidin (Supplementary Table 3). For labeling of Rab proteins (Supplementary Table 3), an extra primary antibody step was performed after fixation instead of labeling cytoskeletal F-actin. DAPI was used as a nuclear counterstain. Images of fluorescent-labeled markers were obtained with a Leica TCS SP8 *X* microscope and a 63x oil immersion objective lens. 3D images were obtained by scanning multiple *XY* planes in the *Z* direction with a depth of ± 10 µm. Serial pictures along the *Z*-axis were combined to create a stacked *XY* image. The total internalized VE-cadherin area was determined by using ImageJ 1.47v.

### Morpholino injection in developing zebrafish larvae

Zebrafish (Danio rerio) were maintained under standard laboratory conditions. Morpholinos (MO) against the zebrafish orthologue of CMTM4 were obtained from Gene Tools (Philomath, USA) and suspended in Danieau buffer [58 mM NaCl, 0.7 mM KCl, 0.4 mM MgSO4, 0.6 mM Ca(NO3)2, 5.0 mM HEPES pH 7.6 containing 0.2% phenol red]. Different doses of the MO were injected into single-cell stage zebrafish embryos as previously described [21]. The sequences are listed in Supplementary Table 4.

### Flow cytometry

CMTM4-silenced HUVECs were seeded at a density of 50 × 10^3^ cells/well in co-culture medium. After 24, 48, and 72 h, cells were harvested for proliferation and apoptosis analysis. Each harvested sample was stained with Propidium Iodide (PI) and Annexin V (BD Pharmingen) according to the manufacturer’s protocol and analyzed by flow cytometry (BD Facs Canto). Cells were counted for 2 min at medium speed to determine the average cell amount. Cell cycle analysis was conducted after the cells were cultured for 48 h at 37 °C in 5% CO2 and fixed overnight in 70% ethanol at 4 °C. Cells were washed with ice-cold PBS and were stained with PI and treated with 0.5 mg/ml RNase for 30 min at 37 °C before analysis by flow cytometry.

### Migration assay

HUVECs were seeded at a density of 75 × 10^3^ cells/well in a 24-well plate and after 24 h. EC migration was assessed 24-h post-transfection by scratching the HUVEC layer using a 200-µl-pipette tip. Loose cells were removed by a PBS wash. Images were taken at *t* = 0, *t* = 4, and *t* = 8 h, after which the scratch area reduction was determined using Adobe Photoshop CS6.

### Intracellular immunostaining

HUVECs were seeded at a density of 10 × 10^4^ cells/well 24 h prior to transfection. Cells were fixed 48-h post-transfection with 4% paraformaldehyde for 30 min, followed by a blocking and permeabilization step for 30 min in PBS with 1% BSA and 0.5% Triton-X. This was followed by incubation with a primary and secondary antibody (Supplementary Table 3) at room temperature for 1.5 and 1 h, respectively. Cytoskeletal F-actin was labeled with rhodamine phalloidin and DAPI was used as a nuclear counterstaining. Images of fluorescent-labeled markers were obtained with a Leica TCS SP8 X microscope and a 63x oil immersion objective lens. For quantification of the total area, 3D images were obtained by scanning multiple *XY* planes in the *Z* direction with a depth of ± 10 µm. Serial pictures along the *Z*-axis were combined to create a stacked *XY* image that was further analyzed using ImageJ 1.47v.

### Transendothelial resistance measurements

HUVECs were transfected with CMTM4 siRNA or adenovirus 24 h prior to seeding on a 0.1% gelatin permeable filter insert (0.4 µm pore, Falcon). Before the experiment, the resistance (*R*_blank_) was measured by placing unseeded inserts in an Endohm-SNAP chamber filled with 5 ml of EGM-2 medium (World Precision Instruments, Berlin). The chamber was coupled to an EVOMX resistance meter (World Precision Instruments, Berlin). The transendothelial electrical resistance (TEER) was measured daily to monitor resistance buildup during growth towards full confluence. At day 2, confluent monolayers were treated with 1 U/ml thrombin for 20 min during which the TEER was measured every 5 min. After 20 min, the thrombin solution was washed away and replaced with normal medium. TEER was measured every 15 min during the restoration phase for 2 h.

### Statistical analysis

The statistical analyses were performed using Graphpad Prism version 7.02. To test if values came from a Gaussian distribution, the D’Agostino–Pearson omnibus or Shapiro–Wilk normality test were used. The unpaired t-test and the ordinary one-way ANOVA test were used if the values were normally distributed. In case the values did not pass the normality test, either the Mann–Whitney test or Kruskal–Wallis were used as non-parametric tests. *P* ≤ 0.05 was accepted as statistically significant. Values are shown as mean ± SEM.

## Results

### CMTM4 expression is essential for vascular growth in an in vitro 3D angiogenesis assay

**Fig. 1:**
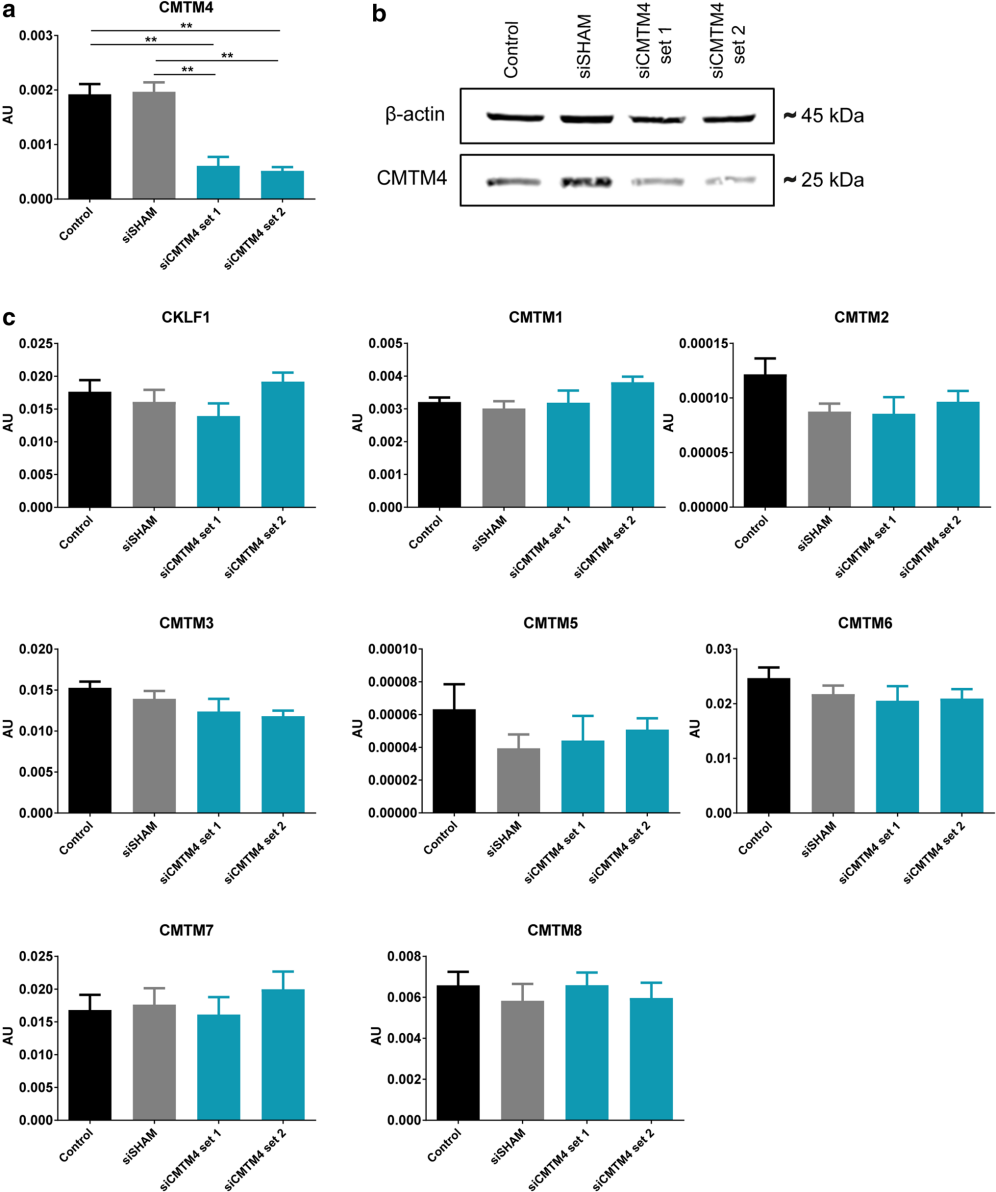
CMTM4-targeting siRNA induces significant and specific silencing of CMTM4 expression. **a** Gene expression levels of CMTM4 in HUVECs transfected with CMTM4-targeting siRNA (siCMTM4), non-targeting siRNA (siSHAM), and in non-transfected HUVECs (control). **b** Representative Western blot of CMTM4 and *β*-actin protein levels in siCMTM4, siSHAM, and control HUVECs. Shown is a representative blot of 3 blots. **c** Gene expression levels of CMTM family members in siCMTM4, siSHAM, and control HUVECs. For **a** and **c**, gene expression levels were normalized to β-actin (AU); values are mean ± SEM, **P < 0.01; *N* ≥ 8 qPCRs

The function of CMTM4 was investigated in vitro using siRNA-mediated silencing in HUVECs. Both qPCR and Western blot analysis confirmed efficient silencing of CMTM4 in cells transfected with two different siRNA sets specific for CMTM4 (siCMTM4 set 1 and set 2), compared to untreated cells (control) and cells transfected with a pool of non-targeting siRNA sequences (siSHAM) (Fig. 1a, b). The mRNA levels of the other CMTM family members were not significantly downregulated by both CMTM4 targeting siRNA sets when comparing siCMTM4 versus siSHAM, indicating that the siRNA-mediated silencing was specific for CMTM4 (Fig. 1c).

**Fig. 2:**
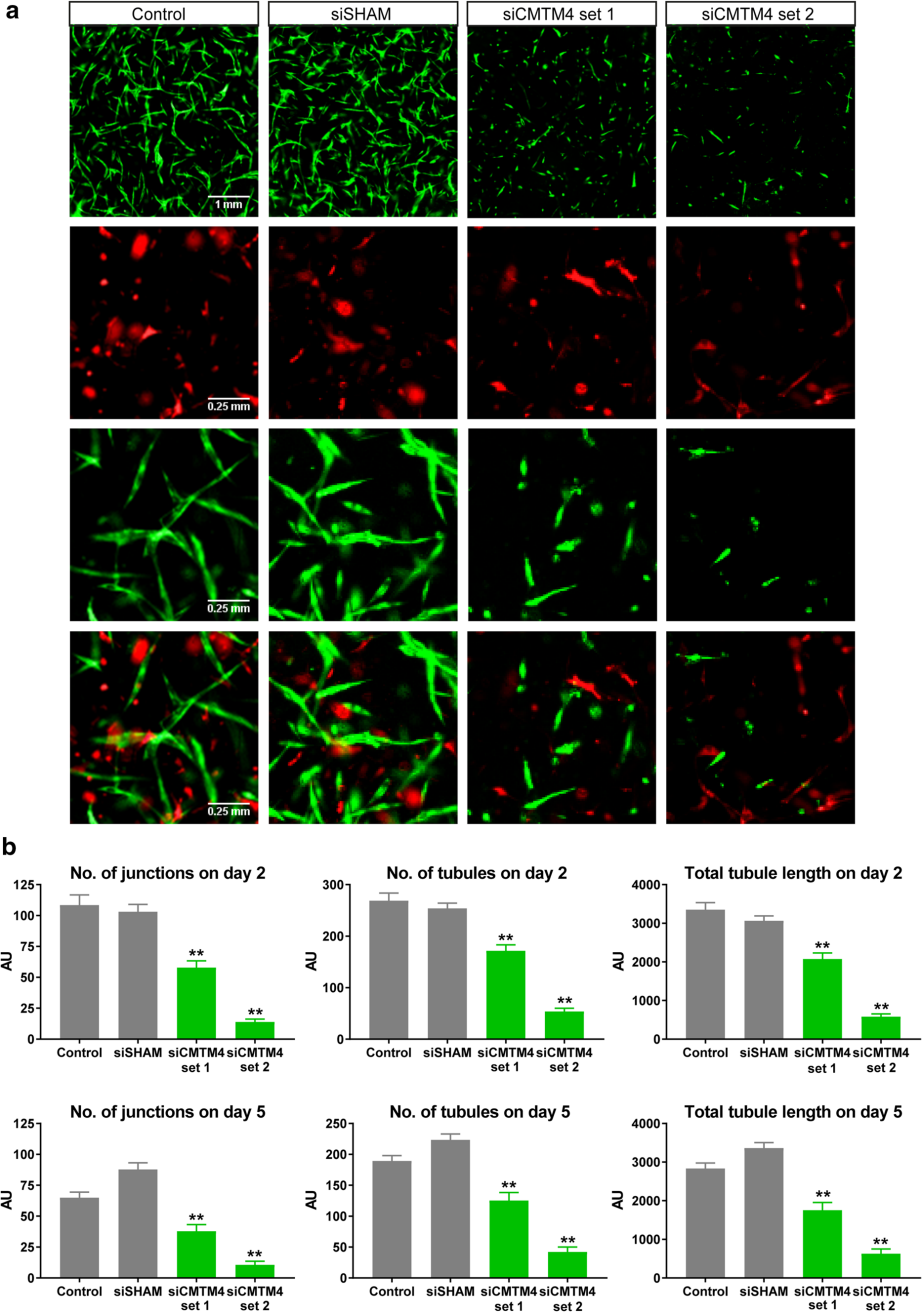
CMTM4 silencing is essential for vascular growth in vitro. **a** Representative immunofluorescent images taken at ×2 magnification (upper row) and zoomed-in images (three lower rows) of GFP-labeled HUVECs (green) and dsRED-labeled pericytes (red) cultured for 5 days in a 3D collagen matrix, in which the HUVECs-GFP were transfected with either CMTM4-targeting siRNA (siCMTM4) or non-targeting siRNA (siSHAM) or not transfected (control). Scale bar represents 1 mm for the upper row and 0.25 mm for the three lower rows. **b** Quantification of the number of junctions, number of tubules, and total tubule length of siCMTM4, siSHAM, and control HUVECs at day 2 and 5 of co-culture. Values are mean ± SEM, **P < 0.01 compared to all other conditions; *N* ≥ 6 co-cultures, ± 7 images each

Next, the angiogenic potential of these CMTM4-silenced HUVECs was assessed in vitro in a 3D angiogenesis assay developed for studying the formation of lumenized micro-capillary structures [22]. In this assay, GFP-expressing HUVECs and dsRED-expressing pericytes directly interact in a collagen type I matrix. After 1 day of co-culture, EC sprouting and tubule formation can be observed and these neovascular structures are stabilized by perivascular recruitment of pericytes. After 5 days, micro-capillaries with distinct luminal areas and pericyte coverage can be observed. Imaging and quantification of these vascular structures was conducted at day 2 and 5. This assay has been well validated in previous studies [23–25]. Endothelial silencing of CMTM4 severely impaired the formation of neovascular structures in the 3D angiogenesis assay when compared to control and siSHAM (Fig. 2a). Quantification revealed a significant reduction in the number of junctions, the number of tubules, and the total tubule length of HUVECs after both 2 and 5 days of co-culture (Fig. 2b). In contrast, CMTM4 silencing in pericytes did not affect tubule formation at 2 or 5 days of co-culture (Supplementary Fig.1a–d).

### Silencing of CMTM4 in developing zebrafish larvae inhibits intersomitic vessel growth

**Fig. 3:**
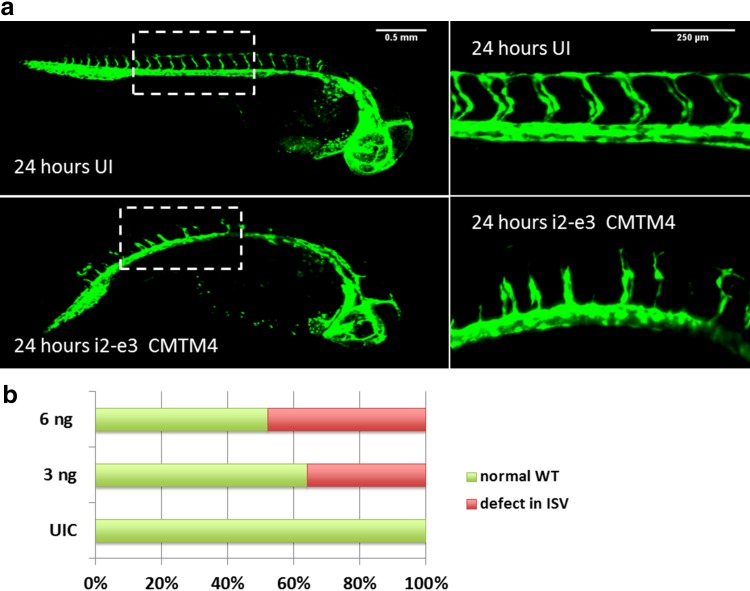
Morpholino silencing of CMTM4 in developing zebrafish larvae induces defects in intersomitic vessels. **a** Tg(fli1:eGFP)_y1_ larvae at 24 h post fertilization, anterior to the right, lateral view. Defects in intersomitic vessel (ISV) formation was observed in the trunk region in specimens injected with morpholinos targeting the splice site of i2-e3 of CMTM4 (indicated as i2-e3 CMTM4) compared with uninjected (UI) controls. The vasculature is highlighted by eGFP (green). Left-hand panels show ×2 magnification images with 0.5 mm scale bar, right-hand panels show 10x images with 0.25 mm scale bar. **b** Quantification of ISV defect phenotype versus wildtype (WT) phenotype in i2-e3 injected group at different doses versus UI controls (UIC). Data represent percentage of counted larvae (~ 100 per group)

In order to evaluate CMTM4 function during in vivo angiogenesis, the zebrafish orthologue of the gene was silenced in developing larvae of the transgenic zebrafish line Tg(fli1:eGFP)_y1_ by morpholino knockdown. CMTM4 is highly conserved in the zebrafish genome and two morpholinos were designed to target the CMTM4 zebrafish orthologue with knockdown based on the splice modification principle of the pre-mRNA target. Injection with a morpholino construct that targeted the splice site located on CMTM4 intron 2-exon 3 (i2-e3) affected intersomitic vessel (ISV) formation versus uninjected larvae at 24 h post fertilization (Fig. 3a). Phenotype quantification showed in the 3 ng i2-e3 morpholino group that 36% of larvae displayed defects in ISV formation versus 0% in the control group. Injection with 6 ng i2-e3 increased the number of larvae with affected ISVs to 48% (Fig. 3b). In line with these findings, injection of the second morpholino construct that targeted the splice site on CMTM4 intron 1-exon 2 (i1-e2) affected ISV formation in 56% of the larvae in the i1-e2 group versus 0% in the control population (Supplementary Fig. 2a, b). These data clearly demonstrate a negative effect of CMTM4 silencing on angiogenesis in developing zebrafish larvae in vivo.

### Effect of CMTM4 on cell cycle progression, migration, and proliferation

Previous studies in HeLa and clear cell renal cell carcinoma cells imply that CMTM4 functions as a tumor suppressive gene, with expression of CMTM4 leading to cell migration and inhibition of cell proliferation via G2/M phase accumulation [14, 15]. We investigated the effects of CMTM4 silencing in HUVECs on these parameters. CMTM4 silencing using either siRNA set 1 or 2 did not affect cell proliferation as shown by comparison with siSHAM-treated and non-treated control groups based on cell count and PI analysis of the G0/G1-phase and G2 phase in fixed cells (Supplementary Fig. 3a, b). Migration capacity was also assessed by scratch assay and showed a significant decrease in response to CMTM4 silencing by set 2, but not by set 1, in HUVECs compared to siSHAM-treated or non-treated controls after 4 and 8 h of migration (Supplementary Fig. 3c–e).

**Fig. 4:**
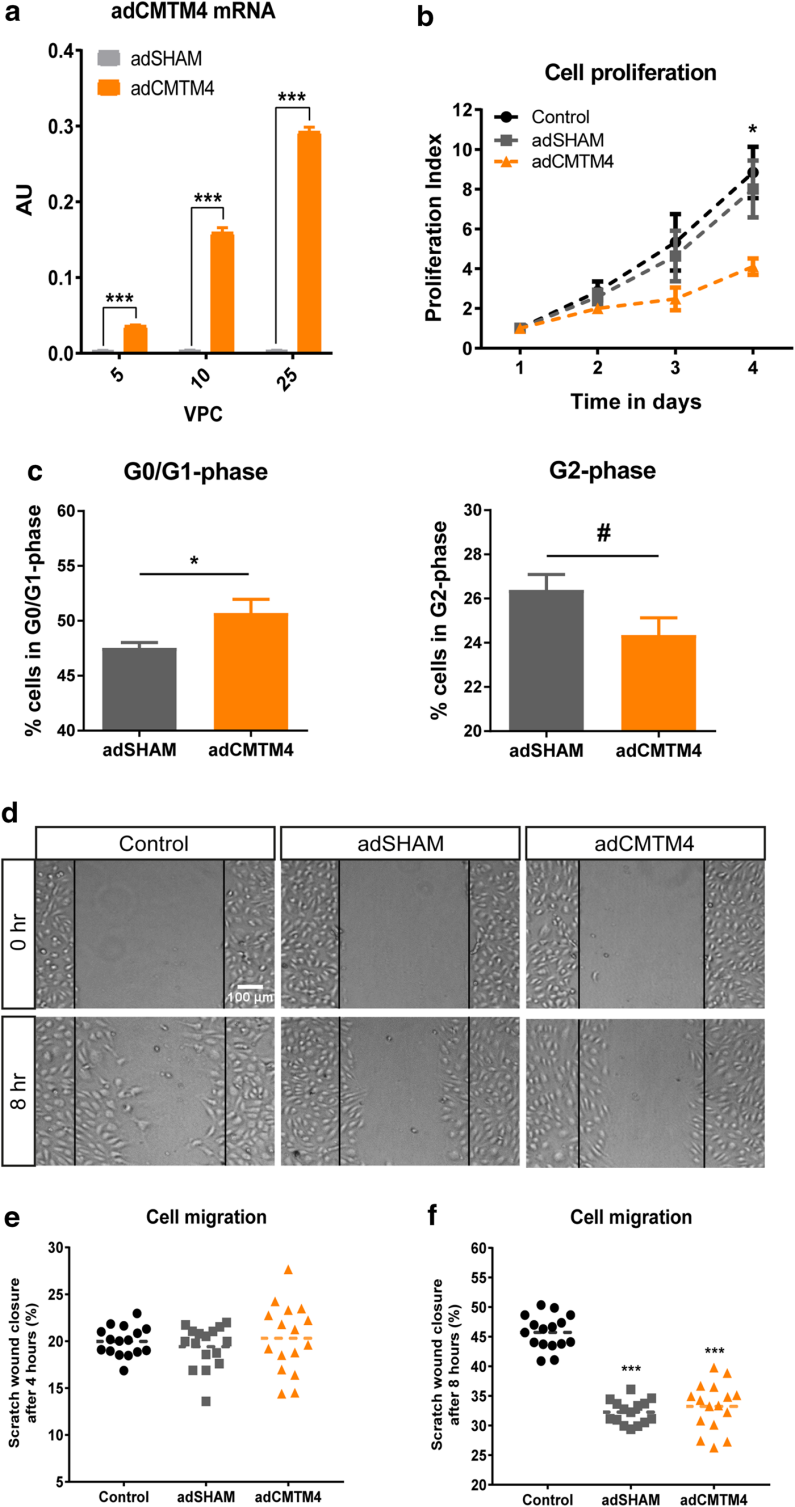
CMTM4 overexpression limits endothelial cell proliferation, but does not affect cell migration. **a** Quantitative polymerase chain reaction (qPCR) of human CMTM4 expression in HUVECs transfected with different (5, 10, 25) virus particles per cell (VPC) of adenovirus containing an expression vector for CMTM4 cDNA (adCMTM4) or with sham virus containing an empty expression vector (adSHAM). Shown are target gene/house-keeping gene (β actin) ratios (AU). Mean ± SEM, ***P < 0.001 in comparison between adSHAM and adCMTM4 group of equal VPC; *N* = 4 qPCRs. **b** The number of cells counted at day of seeding (day 1), and at day 2, 3, and 4 of cell proliferation in adCMTM4 HUVECs compared with adSHAM and non-transfected controls. Shown is mean ± SEM, *N* = 6; *P < 0.05. **c** Bar graphs showing the % of G0/G1 and G2 phase cells at day 2 after transfection in adCMTM4 versus adSHAM HUVECs and non-transfected controls. Shown is mean ± SEM, *P < 0.05, ^#^*P* < 0.10; *N* = 4. **d** Representative brightfield microscope images (x4 magnification) of a scratch migration assay after 8 h of migration of adCMTM4 HUVECs compared to adSHAM and non-treated controls. Scale bar represents 100 µm. Bar graphs of the quantified results of the scratch migration assay showing the % area within the scratched region covered by adCMTM4 HUVECs compared to adSHAM and non-treated controls after 4 h (**e**) and 8 h (**f**). Shown is a dot plot with the distribution and mean, ***P < 0.001; *N* = 2, 8 wells each

Next, we investigated the effects of CMTM4 overexpression on the same parameters. HUVECs were transfected with a recombinant adenovirus that encodes for human CMTM4 cDNA (adCMTM4). qPCR analysis validated significant transgenic expression of CMTM4 in HUVECs compared to HUVECs transfected with sham virus (adSHAM) (Fig. 4a). In contrast with CMTM4 silencing, cell count and cell cycle progression were decreased in adCMTM4 HUVECs versus adSHAM or non-transfected groups, as shown by reduced cell count and an increase of cells in G0/G1 versus G2 phase (Fig. 4b, c). However, overexpression of CMTM4 did not affect migration capacity of HUVECs (Fig. 4d–f). These findings indicate that increased levels of CMTM4 limit cell proliferation, but do not affect cell migration.

### CMTM4 colocalizes with endocytic vesicle structures and VE-cadherin in adherens junctions

**Fig. 5:**
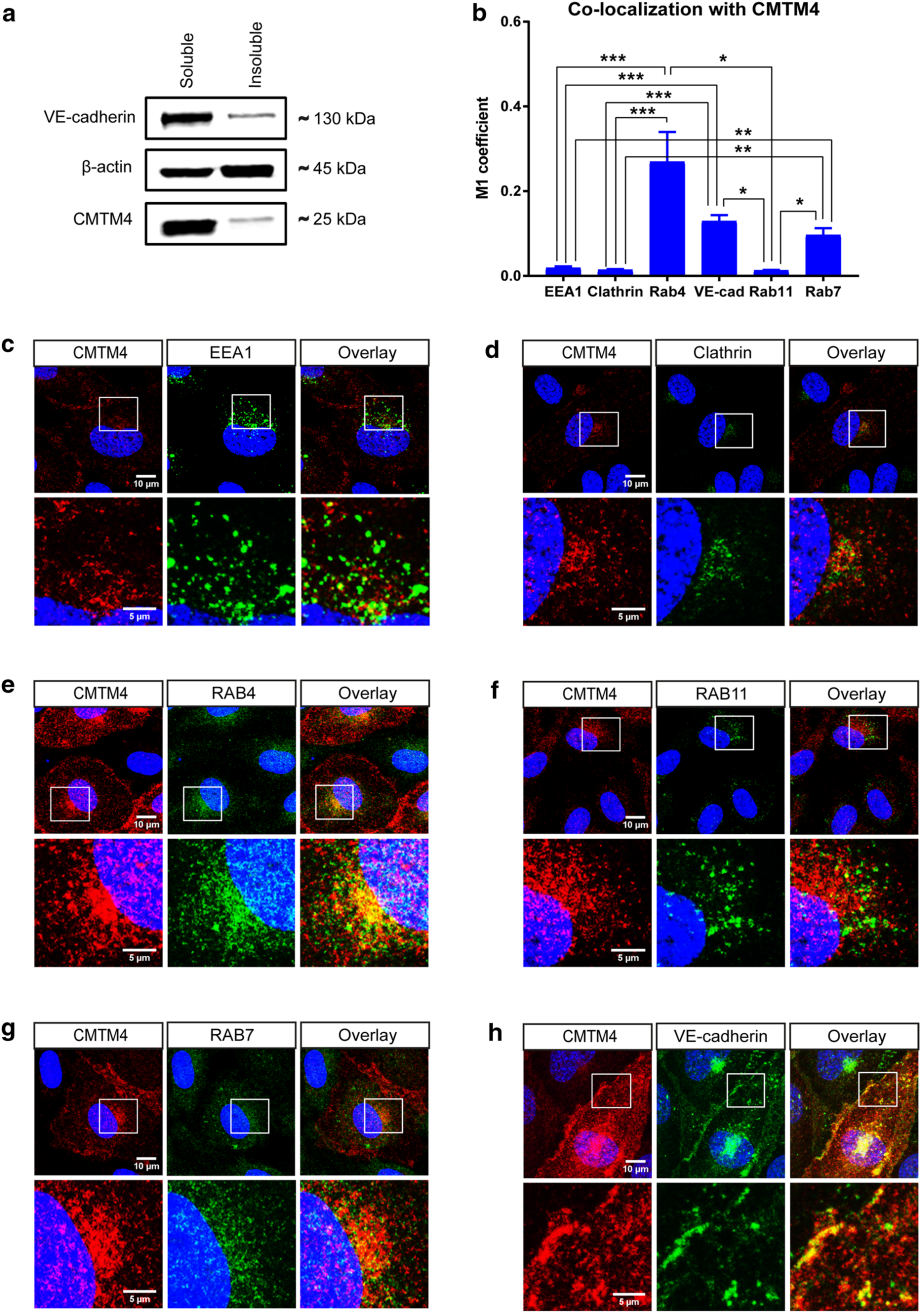

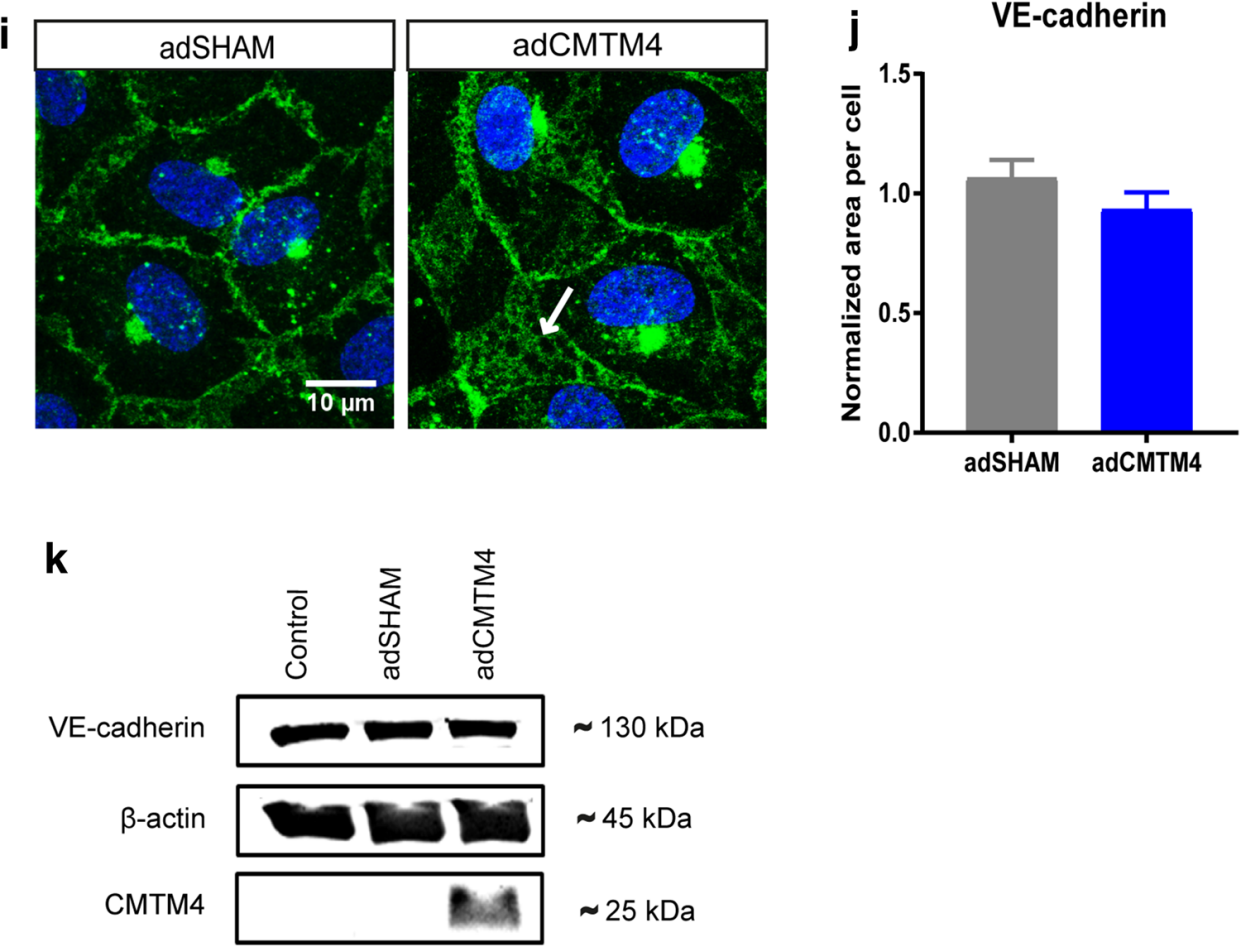
CMTM4 localizes with different vesicle compartments of the intracellular endocytosis pathway and VE-cadherin^+^ adherens junctions. **a** Representative Western blot of CMTM4, β-actin, and VE-cadherin protein levels in the soluble and insoluble cell lysates fraction of HUVECs. **b** Quantification of colocalization of CMTM4 with VE-cadherin and endocytic compartment markers in confocal micrographs based on M1 coefficient. Mean ± SEM, *P < 0.05, **P < 0.01, ***P < 0.001; *N* = 3, 5 fluorescent images each. Representative ×63 magnification (upper row) and zoomed-in (lower row) confocal microscope images of HUVECs transfected with adCMTM4 (VPC10), immunostained for CMTM4 (red) and EEA1 (green) (**c**) Clathrin (green) (**d**), Rab4 (green) (**e**), Rab11 (green) (**f**), Rab7 (green) (**g**), and VE-cadherin (green) (**h**). Colocalization of CMTM4 and endocytic compartment protein markers or VE-cadherin in overlay images is displayed in yellow. Scale bars represent 10 µm for the upper rows and 5 µm for the lower rows. **i** Representative immunofluorescence microscope images of VE-cadherin (green) in adSHAM and adCMTM4 HUVECs (VPC10). White arrow marks the altered VE-cadherin junctional pattern into a plaque-like structure. Scale bar represents 10 µm. **j** Quantification of total VE-cadherin^+^ area per cell in adSHAM or adCMTM4-transfected HUVECs (VPC10). Mean ± SEM; *N* = 3, 5 fluorescent Z-stacks each. **k** Representative Western blot bands for total CMTM4, VE-cadherin, and β-actin expression of adCMTM4 and adSHAM HUVECs (VPC5). Shown is a representative blot of 2 blots

In a recent study, we revealed that CMTM3 is mainly localized in the cytosol, where it is involved in regulating the early endocytosis of the AJ protein VE-cadherin [19]. Therefore, we evaluated in this study the intracellular function of CMTM4 in ECs. Western blot analysis demonstrated that CMTM4 was mainly present in the soluble cytosolic fraction and not in the insoluble more actin enriched fraction of the HUVEC lysates (Fig. 5a). Similarly, VE-cadherin was mainly enriched in the soluble fraction (Fig. 5a).

Double immunofluorescent staining of CMTM4 with different markers of intracellular vesicular transport compartments showed limited colocalization with early endosome markers EEA1 and Clathrin (Fig. 5b–d). In contrast, examination of CMTM4 with markers of recycling vesicles showed significant colocalization between CMTM4 and Rab4, but not Rab11 (Fig. 5b, e, f). Furthermore, colocalization between CMTM4 and Rab7, a marker for vesicles destined for the lysosome pathway, was also significantly increased compared to EEA1, clathrin, and Rab11 (Fig. 5b, g). These findings indicated that CMTM4 was colocalized in (Rab4 marked) recycling and (Rab7 marked) lysosome-destined vesicles and may contribute to recycling and lysosome activity in the process of endocytosis. Further analysis of CMTM4 intracellular localization showed cytosolic and cell–cell junction localization of the protein. Dual immunostaining analysis demonstrated that the CMTM4 signal colocalized with VE-cadherin, both at the AJs and in cytosolic vesicle structures (Fig. 5b, h). Transgenic overexpression of CMTM4 in ECs appeared to alter the junctional pattern of VE-cadherin into plaque-like structures (Fig. 5i). Studies have shown that Junction-Associated-Intermittent-Lamellipodia (JAIL) structures appear at established endothelial junctions and induce an overlap with the plasma membrane of the neighboring cell, forming subsequent VE-cadherin plaques that, after retraction of JAIL, are incorporated into the junctions [26, 27]. Overexpression of CMTM4 did not affect the total VE-cadherin^+^ area (Fig. 5j). Similarly, Western blot analysis of adCMTM4 or adSHAM-transfected HUVECs showed no difference in total protein concentration of VE-cadherin (Fig. 5k).

**Fig. 6:**
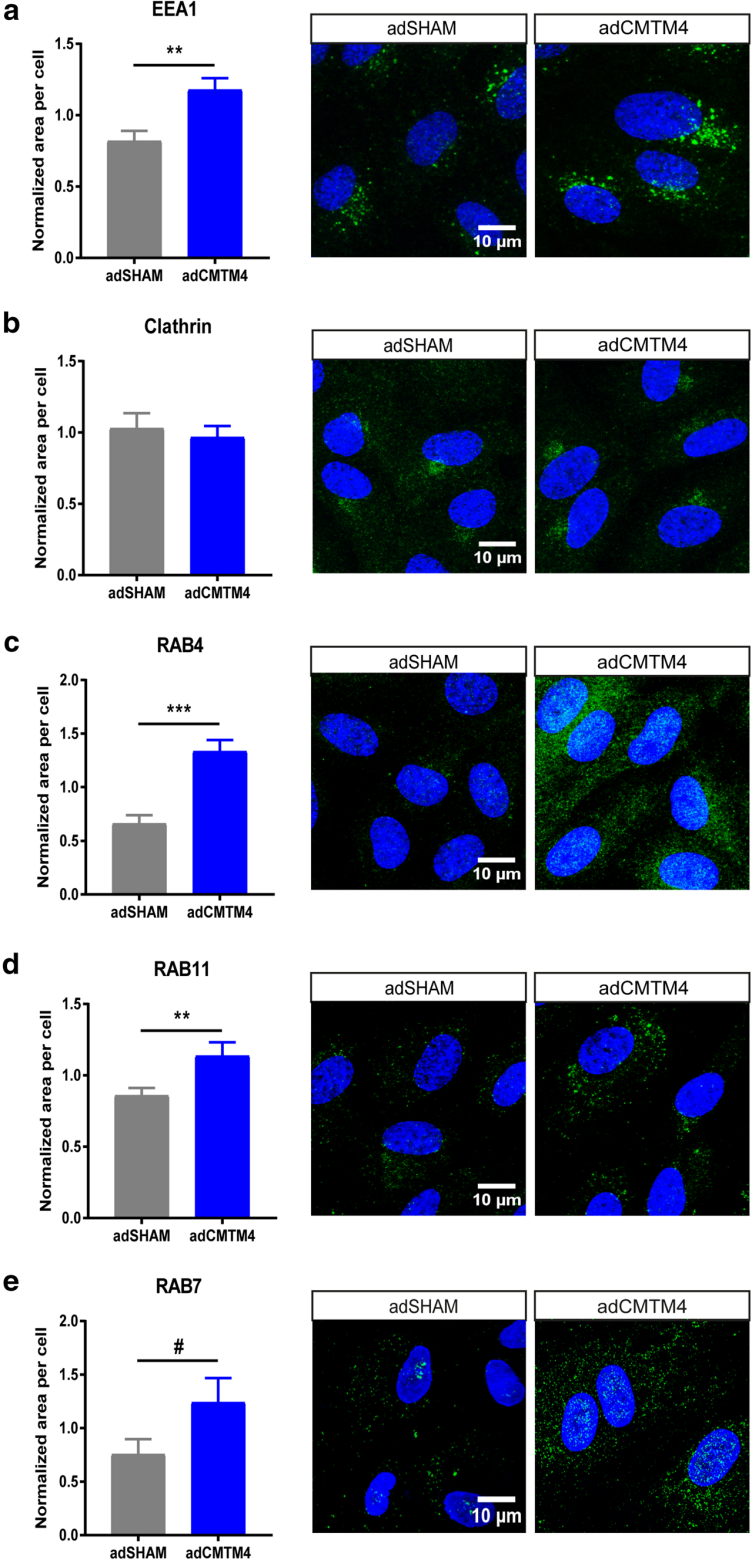
CMTM4 overexpression significantly upregulates the endothelial endocytic pathway. Quantification of area per cell in adCMTM4 and adSHAM-transfected HUVECs (VPC10). Representative confocal micrographs (×63 magnification) of HUVECs transfected with adCMTM4 and adSHAM (VPC10), immunostained (green) for EEA1 (**a**), Clathrin (**b**), Rab4 (**c**), Rab11 (**d**), and Rab7 (**e**). For all data displayed, mean ± SEM, ^#^*P* < 0.1, **P < 0.01, ***P < 0.001; *N* = 3, 5 Z-stacks each; scale bars represent 10 µm

Quantification of EEA1, Clathrin, Rab4, Rab11, and Rab7 immunofluorescence signals showed that EEA1^+^, Rab4^+^, and Rab11^+^ vesicles were significantly increased in adCMTM4 versus adSHAM-treated HUVECs and a similar trend was visible for Rab7^+^ vesicles. In contrast, the amount of Clathrin^+^ vesicles was not affected (Fig. 6a–e). These data indicate that CMTM4 enhances endocytosis processes in ECs.

### CMTM4 regulates VE-cadherin endocytosis and promotes endothelial barrier function of endothelial adherens junctions

**Fig. 7:**
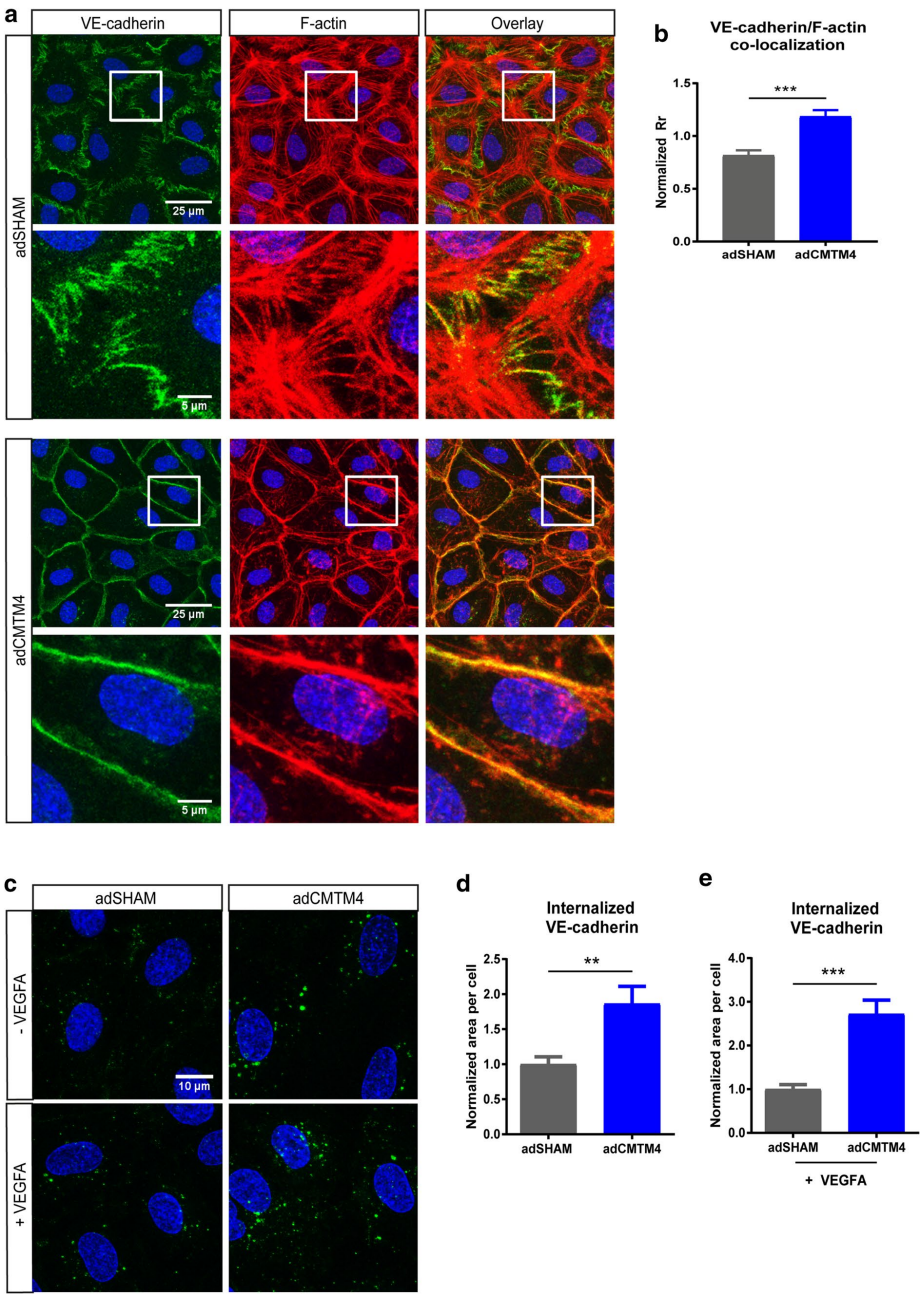

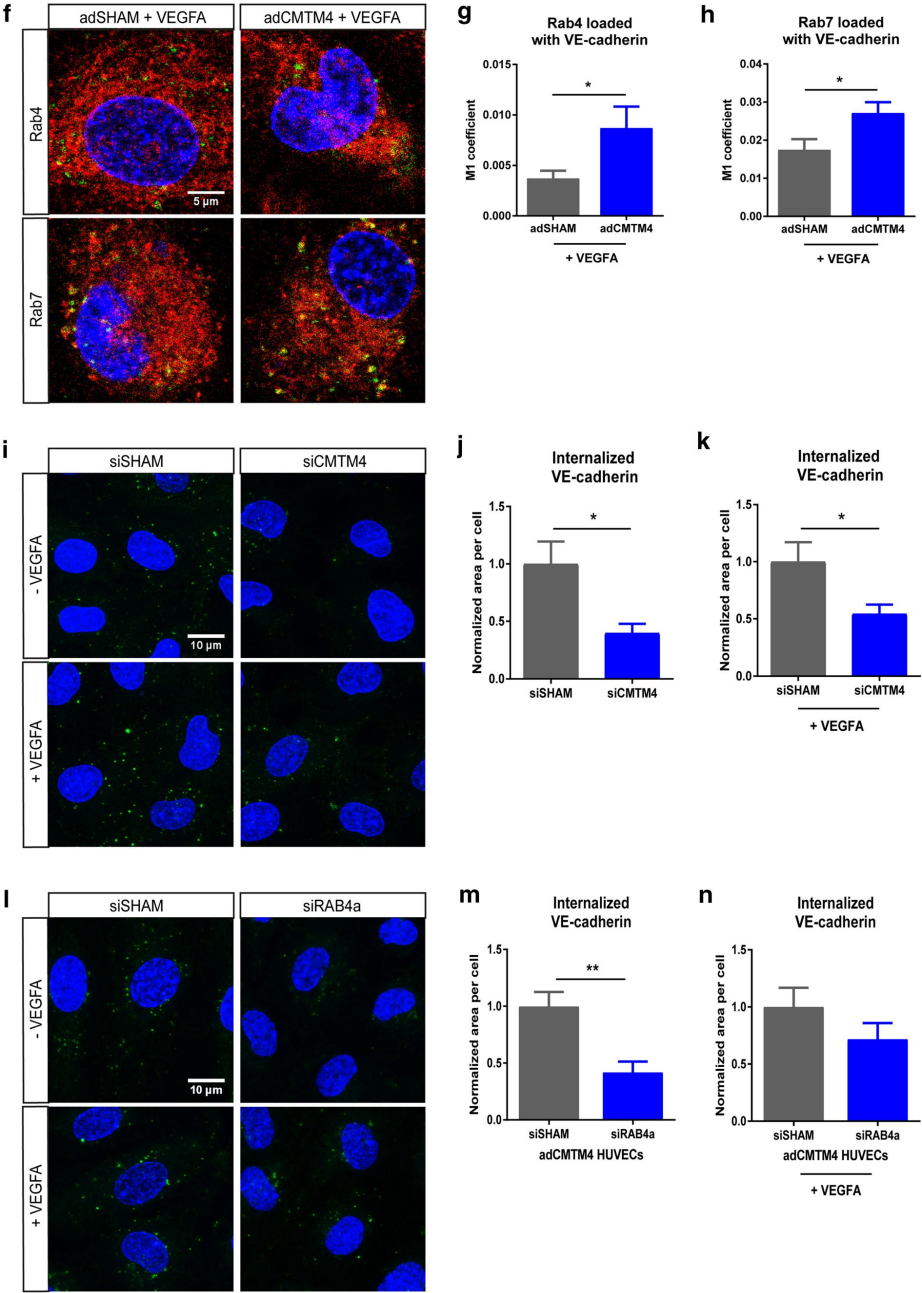
CMTM4 upregulates endocytosis and subsequent recycling of VE-cadherin. **a** Representative ×63 magnification (upper row) and zoomed-in (lower row) confocal microscope images of confluent HUVEC cultures that were immunostained for F-actin (red) and VE-cadherin (green) in adSHAM and adCMTM4-transfected cells (VPC10) that were exposed to low-temperature shock (1 h at 4 °C) followed by 1-h incubation at 37 °C. Adherens junctions were quicker restored in adCMTM4 condition versus adSHAM conditions (zigzagged VE-cadherin signal in adSHAM versus linear VE-cadherin junctions). Colocalization signal in overlay is in yellow. Scale bars represent 25 µm for the upper rows and 5 µm for the lower rows. **b** Quantification of the colocalization signal between VE-cadherin and F-actin in confocal micrographs based on normalized Pearson correlation coefficient (Rr). Mean ± SEM, ***P < 0.001 versus adSHAM; *N* = 4, 5 Z-stacks each. **c** Representative confocal micrographs (×63 magnification) of confluent adSHAM and adCMTM4 cultures (VPC10) labeled with VE-cadherin antibodies at 4 °C to track VE-cadherin movement, followed by 37 °C incubation for 1 h with and without VEGFA stimulation. Surface bound VE-cadherin antibodies were removed by an acid-wash, before proceeding with immunostaining for visualization of internalized VE-cadherin (green). Scale bar represents 10 µm. Quantification of internalized VE-cadherin area per cell in adSHAM and adCMTM4 HUVECs without VEGFA (**d**) and with VEGFA (**e**) stimulation. Mean ± SEM, **P < 0.01, ***P < 0.001 versus adSHAM; *N* = 4, 5 Z-stacks each. **f** Representative zoomed-in confocal micrographs (×63 magnification) of confluent adSHAM and adCMTM4 cultures (VPC10) labeled with VE-cadherin antibodies at 4 °C to track VE-cadherin movement, followed by 37 °C incubation for 1 h with and without VEGFA stimulation. Surface bound VE-cadherin antibodies were removed by an acid-wash, before proceeding with immunostaining for visualization of internalized VE-cadherin (green) and either Rab4 or Rab7 (red). Scale bar represents 5 µm. Quantification of colocalization of either Rab4 (**g**) or Rab7 (**h**) with VE-cadherin in confocal micrographs based on M1 coefficient. Mean ± SEM, *P < 0.05; *N* = 2, 5 fluorescent images each. **i** Representative confocal micrographs (×63 magnification) of confluent siSHAM and siCMTM4 cultures labeled with VE-cadherin antibodies at 4 °C to track VE-cadherin movement, followed by 37 °C incubation for 1 h with and without VEGFA stimulation. Surface bound VE-cadherin antibodies were removed by an acid-wash, before proceeding with immunostaining for visualization of internalized VE-cadherin (green). Scale bar represents 10 µm. Quantification of internalized VE-cadherin area per cell in siSHAM and siCMTM4 HUVECs without VEGFA (**j**) and with VEGFA (**k**) stimulation. Mean ± SEM, *P < 0.05 versus adSHAM; *N* = 2, 5 Z-stacks each. **l** Representative confocal micrographs (×63 magnification) of confluent siSHAM HUVECs and HUVECs transfected with Rab4a-targeting siRNA (siRAB4a) labeled with VE-cadherin antibodies at 4 °C to track VE-cadherin movement, followed by 37 °C incubation for 1 h with and without VEGFA stimulation. Surface bound VE-cadherin antibodies were removed by an acid-wash, before proceeding with immunostaining for visualization of internalized VE-cadherin (green). Scale bar represents 10 µm. Quantification of internalized VE-cadherin area per cell in siSHAM and siRAB4a HUVECs without VEGFA (**m**) and with VEGFA (**n**) stimulation. Mean ± SEM, **P < 0.01 versus adSHAM; *N* = 2, 7 Z-stacks each

The putative role of CMTM4 in endocytosis processing of VE-cadherin was evaluated with an internalization assay, where confluent HUVECs transfected with adSHAM or adCMTM4 were exposed to a low-temperature shock (1 h at 4 °C) followed by an 1-h incubation at 37 °C. Assessment of VE-cadherin in relation to the actin cytoskeleton showed a significant increase in colocalization in adCMTM4-treated versus adSHAM confluent HUVEC monolayers, as demonstrated by an increase in Pearson correlation coefficient (Fig. 7a, b). This finding indicates that CMTM4 promotes VE-cadherin connections with the actin cytoskeleton that are vital for AJ stabilization.

Next, the role of CMTM4 in VE-cadherin endocytosis was assessed. For this, confluent adSHAM or adCMTM4 HUVECs were incubated with VE-cadherin antibody at 4 °C before an acid-wash treatment to remove all extracellular VE-cadherin signal. Subsequent analysis following 1-h incubation at 37 °C with and without VEGFA stimulation showed a significant increase of internalized VE-cadherin in adCMTM4 versus adSHAM conditions (Fig. 7c–e). The internalized VE-cadherin upon VEGF stimulation was present in both Rab4^+^ and Rab7^+^ vesicles with significantly more Rab4^+^ and Rab7^+^ vesicles loaded with VE-cadherin in adCMTM4 HUVECs when compared to adSHAM HUVECs (Fig. 7f–h). In line with these findings, siRNA-mediated knockdown of CMTM4 significantly decreased the signal of internalized VE-cadherin in siCMTM4-treated HUVECs with and without VEGFA stimulation (Fig. 7i–k). This effect of CMTM4 was dependent on Rab4, since adCMTM4 HUVECs transfected with siRNA specific for Rab4a (siRAB4a) also showed significantly less internalized VE-cadherin when compared to siSHAM HUVECs. The trend was still visible under VEGF stimulation (Fig. 7l–n and Supplementary Fig. 4a).

**Fig. 8:**
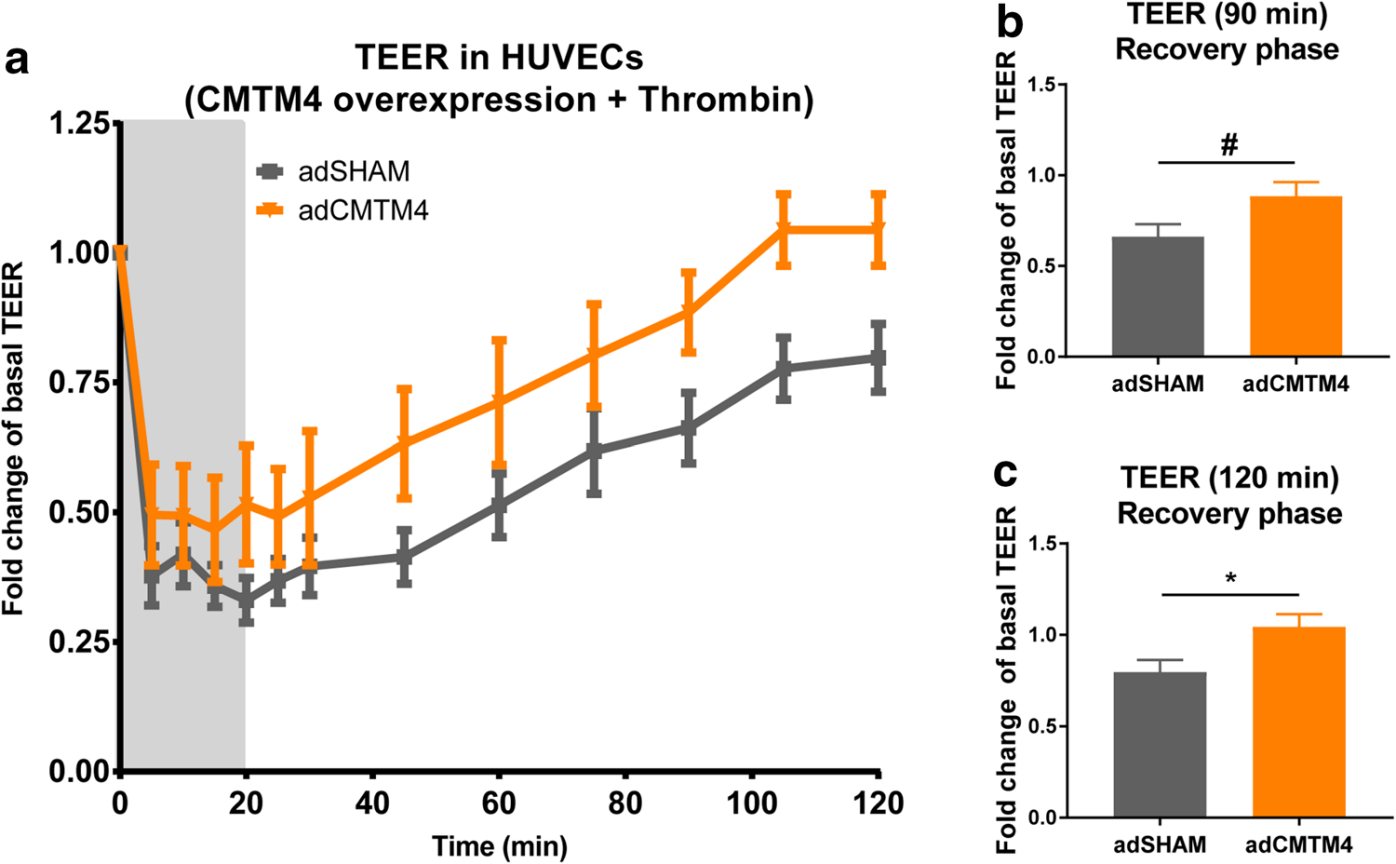
CMTM4 promotes restoration of endothelial electric resistance in thrombin-induced response. **a** Thrombin response (presented in fold change compared with basal resistance of non-transfected group: Y axes) of confluent HUVEC monolayers during (0–20 min) and after (20–120 min) thrombin (1 U/mL) stimulation in adCMTM4 and adSHAM conditions. At 0 min, thrombin was added and after 20 min thrombin was removed (gray area). Mean ± SEM. *N* ≥ 4 per time series. Increase in resistance during recovery phase at 90 min (**b**) and 120 min (**c**) in adSHAM and adCMTM4 groups. Mean ± SEM, ^#^*P* < 0.10 versus adSHAM; *N* ≥ 6

Findings so far indicate that CMTM4 could regulate the bio-availability of VE-cadherin at cell–cell AJs, which implies that it could impact endothelial barrier function. We used transendothelial electric resistance (TEER) measurements to evaluate the response of HUVEC monolayers transfected with adSHAM or adCMTM4 to thrombin-induced junction disruption. Basal levels (before thrombin stimulation) of TEER were not affected by CMTM4 overexpression. However, the recovery of TEER post-thrombin stimulation was significantly increased in adCMTM4 conditions compared to adSHAM (Fig. 8a–c). In line with these findings, basal TEER levels were similarly not affected by siRNA-mediated silencing of CMTM4, but TEER recovery after thrombin removal was significantly decreased in siCMTM4 versus siSHAM conditions (Supplementary Fig. 4a, b).

## Discussion

In this study, we investigated the angiogenic potential of CMTM4 and evaluated in which molecular pathways CMTM4 is involved in ECs. The most important findings of this study are as follows: (1) CMTM4 silencing impairs vascular growth in an in vitro 3D angiogenesis assay. (2) Silencing of CMTM4 in developing zebrafish larvae inhibits intersomitic vessel growth. (3) Intracellular staining reveals that CMTM4 colocalizes with Rab4^+^ and Rab7^+^ vesicles, and with both membrane-bound and internalized VE-cadherin. (4) CMTM4 enhances the endothelial endocytic pathway, in particular the Rab4 recycling pathway. (5) CMTM4 also enhances VE-cadherin internalization and increases endothelial barrier function recovery.

Several previous studies have revealed that CMTM family members can prevent growth and invasion of different cancer cell types [11–18]. CMTM4 has been reported to be frequently downregulated in clear cell renal cell carcinoma where it functions as a tumor suppressor. Thus, restoration of CMTM4 suppresses the tumorigenicity of these cancer cells, whereas knockdown of CMTM4 leads to enhanced tumor growth [14]. Furthermore, overexpression of CMTM4 inhibits HeLa cell growth via G2/M phase accumulation [15]. In a recent study, we demonstrated that CMTM3 has a specialized function in ECs, where it is involved in the regulation of early endocytosis of VE-cadherin during vascular growth [19]. However, the putative function of CMTM4 in endothelial endocytosis and the contribution of CMTM4 to the regulation of vascular permeability have not been elucidated to date.

In the present study, we demonstrate that CMTM4 plays an important role in angiogenesis. Loss of CMTM4, as shown in vitro with a 3D vascular assay and in vivo with zebrafish larvae, results in a significant reduction of vascular sprouting. ECs can actively modulate their junctional adhesive strength by regulating the amount of bioavailable VE-cadherin on the cell membrane. A reduction in membrane-bound VE-cadherin will result in microvascular destabilization that is required for EC migration and vascular sprouting. Vascular sprouting from pre-existing vessels is mediated by VEGFA by stimulating the endocytosis of VE-cadherin [9]. Similar to CMTM3, we have shown that the angiogenic capacity of CMTM4 is associated with the regulation of cell surface VE-cadherin. CMTM4 overexpression promotes the internalization of VE-cadherin, both at basal levels and in response to VEGFA stimulation. Vice versa, CMTM4 knockdown inhibits VE-cadherin internalization. In line with our findings, it has been shown that a reduction of VE-cadherin both in vitro and in vivo increases vascular permeability and stimulates vascular sprouting [4, 28].

After these initial stages of neovessel formation, VE-cadherin is eventually required for the formation of a stable vasculature by preventing disassembly. This is demonstrated in VE-cadherin-deficient mice, who suffer from severe vascular defects, resulting in death at mid-gestation [5, 6]. In line with those observations, our data indicate that CMTM4 overexpression promotes VE-cadherin connections with the actin cytoskeleton that are vital for AJs stabilization. The VE-cadherin/catenin complex interacts with actin filaments to ensure adequate junction adhesion and barrier function. Usually, the boundaries between adjacent ECs display a stable linear morphology and are regulated by circumferential actin filaments. However, endothelial AJs are highly dynamic and reorganize continuously in response to external stimuli [27]. These linear cell–cell junctions can convert into dynamic discontinuous cell–cell junctions, characterized by the appearance of stress fibers and zigzagged junctions where VE-cadherin aligns with the end of these stress fibers. Discontinuous junctions are characteristic for an activated and permeable endothelium [29]. Our data indicate that AJs are quickly restored in adCMTM4 HUVECs after a cold stimulus, showing a stable resting condition pattern with linear VE-cadherin junctions that colocalize with the circumferential actin filaments. adSHAM HUVECs appear to be at a different stage of cell–cell junction remodeling in response to the cold stimulus, showing a zigzagged VE-cadherin pattern and stress fibers. This discontinuous pattern seen in adSHAM HUVECs will contribute to a rapid increase in endothelial permeability and subsequently a longer recovery phase. In concordance, TEER measurements reveal a fast recovery after thrombin-induced endothelial junction disruption in ECs with overexpression of CMTM4 when compared to sham-treated cells, whereas knockdown of CMTM4 results in a lower recovery rate. Taken together, these results are consistent with the concept that CMTM4 is responsible for a fast turnover of VE-cadherin from the plasma membrane and back, thereby regulating the junctional adhesive strength of ECs and hence the endothelial barrier function.

The turnover of cell surface VE-cadherin involves endocytic trafficking pathways, in which VE-cadherin is transferred into the cytosol in clathrin-coated vesicles and is further sorted for recycling or lysosomal trafficking routes, among others [7]. Cargo proteins can be recycled back to the plasma membrane through two distinct recycling pathways: The Rab4-mediated rapid recycling pathway and the Rab11-mediated slow endosome pathway [30]. Rapid recycling occurs directly from the early endosomal compartment, whereas the slow recycling route involves protein cargo trafficking from either early endosomes or the trans-Golgi network through the pericentriolar recycling endosomal compartment, before returning to the cell surface [31, 32].

In the present study, we demonstrate that CMTM4 strongly colocalizes with Rab4 and VE-cadherin, suggesting that CMTM4 is involved in regulating the rapid recycling of VE-cadherin back to the plasma membrane at AJs. Furthermore, overexpression of CMTM4 significantly increases Rab4^+^ vesicles in the cytosol, but does not increase the total protein amount of VE-cadherin, indicating that CMTM4 regulates the localization, but not the synthesis or degradation of VE-cadherin. It has been shown that overexpression of Rab4 increases the recycling of various receptors back to the cell surface. For example, Rab4 overexpression in CHO (Chinese Hamster Ovary) cells raises the number of transferrin receptors on the cell surface from 20 to 80% [33]. Recycling and activation of the β-Adrenergic receptor is also enhanced by overexpression of Rab4 in cardiac myocytes [34]. Furthermore, Rab4 activation increases the expression of VE-cadherin at the cell surface of lung microvascular endothelial cells (LMVECs), whereas Rab4 inhibition reduces VE-cadherin levels, thereby increasing vascular permeability [35]. More specifically, Rab4 activation enhances EC migration, adhesion, and tube formation [36]. As reported by these studies, silencing of Rab4a in HUVECs overexpressing CMTM4 reduces the internalization of VE-cadherin, indicating that the effect of CMTM4 is dependent on Rab4.

Besides Rab4, we show that CMTM4 overexpression also significantly enhances Rab11^+^ and EEA1^+^ vesicles in the cytosol. EEA1 colocalizes exclusively to early endosomes, from which cargo is further sorted into trafficking routes. Silencing of Rab11a in LMVECs prevents VE-cadherin recycling and expression at the plasma membrane and blocks junctional reannealing after vascular inflammation [37]. CMTM4 does not colocalize with either EEA1 or Rab11, however, our findings do imply that the slow endosome pathway is activated as well in response to CMTM4 overexpression. Sorting endosomes leaving the recycling pathways are organized in different Rab domains, created through the recruitment of specific effector proteins [30]. These recycling endosomes are composed of multiple combinations of Rab proteins, creating distinct endosome populations. Fast recycling is achieved by rapid sorting into Rab4^+^ domains within one endosome and recycling slows down once the cargo arrives to the pericentriolar membranes consisting mainly of Rab4^+^ Rab11^+^ domains [30, 38]. After Rab4 depletion, Rab11^+^ vesicles fuse with the plasma membrane to release the protein cargo [38]. Rab4 is not involved in exocytosis, but acts as a regulator in the formation of recycling vesicles from early endosomes, since removal of Rab4 strongly inhibits the formation of these vesicles [39]. Our findings imply that CMTM4 may function as an effector protein for creating Rab4^+^ domains in rapid and slow recycling endosome populations. CMTM4 may also serve as an adaptor protein for targeting and positioning VE-cadherin in these Rab4^+  ^recycling vesicles, since our findings indicate that CMTM4 is also involved in VE-cadherin endocytosis and recycling.

Instead of recycling back to the plasma membrane, VE-cadherin can also be targeted to the lysosomal trafficking route for degradation. In our dataset, CMTM4 also localizes with Rab7, which is involved in the regulation of late endosomal trafficking to lysosomes [40]. Furthermore, overexpression of CMTM4 enhances Rab7^+^ vesicles in the cytosol, indicating that the lysosomal trafficking route is also activated in response to CMTM4 overexpression. In line with these statements, CMTM4 stimulates the loading of Rab4^+^ and Rab7^+^ trafficking vesicles with internalized VE-cadherin.

In a recent paper, we have shown that family member CMTM3 regulates the endocytosis of VE-cadherin and localizes with EEA1 and Clathrin, both markers of the early endocytic pathway, but not with Rab4 [19]. This generates the perception that the CMTM family may play an important role in the endocytosis and recycling of VE-cadherin in ECs, with each member of the gene family having their own specialized function along the various endocytic trafficking routes. All 9 CMTM family members contain a MARVEL domain, which is characterized by a four transmembrane-helix architecture, that has been associated with proteins involved in vesicle trafficking [41]. In line with this finding, overexpression of CMTM8 in tumor cell lines enhances the endocytosis of the epidermal growth factor receptor (EGFR), whereas knockdown delays endocytosis [42]. We assessed if there is a functional relationship between CMTM4 and CMTM3. HUVECs overexpressing both CMTM4 and CMTM3 trend toward internalizing more VE-cadherin compared to single CMTM overexpression, but it does not reach statistical significance. Both HUVECs with single and double CMTM overexpression do internalize significantly more VE-cadherin than sham-treated cells, implying that CMTM3 and CMTM4 do not hinder each other in their function (Supplementary Fig. 6a, b, g). Normally, CMTM4 localizes both at the cytosol and cell border in ECs, whereas CMTM3 only localizes at the cytosol. This pattern does not change when silencing the other family member, implying that CMTM4 and CMTM3 do not regulate each other’s localization (Supplementary Fig. 6c–f and h). Further studies are required to assess if other family members are involved in the endocytosis regulation of ECs.

In conclusion, we have identified in this study for the first time to our knowledge, a regulatory function for CMTM4 in angiogenesis. CMTM4 plays an important role in the turnover of surface VE-cadherin, thereby mediating endothelial barrier function and controlling vascular sprouting. Further studies are needed to elucidate the exact mechanism by which CMTM4 regulates the endocytosis and trafficking of EC surface proteins. Increasing our understanding of the regulators of endothelial endocytosis could help find novel therapeutic targets for the treatment of various diseases associated with vascular leakage, to block vascular growth in tumors, or to overcome the problem of poor vascularization in tissue engineering.

## Electronic supplementary material

Below is the link to the electronic supplementary material.

Supplementary material 1 (DOCX 4043 KB)
