# Supplementary material for: CMTM4 regulates angiogenesis by promoting cell surface recycling of VE-cadherin to endothelial adherens junctions
Source: Angiogenesis. 2018 Aug 10;22(1):75–93. doi: 10.1007/s10456-018-9638-1 (PMC6510885; doi:10.1007/s10456-018-9638-1)
Supplement: Supplementary file 1 — Supplementary material 1 (DOCX 4043 KB) [file 10456_2018_9638_MOESM1_ESM.docx]

**ANGIOGENESIS**

**Supplementary material**

**CMTM4 regulates angiogenesis by promoting cell surface recycling of VE-cadherin to endothelial adherens junctions**

Ihsan Chrifi*, Laura Louzao-Martinez*, Maarten M. Brandt, Christian G.M. van Dijk, Petra E. Burgisser, Changbin Zhu MD, Johan M. Kros MD, Marianne C. Verhaar, Dirk J. Duncker, Caroline Cheng.

** These authors contributed equally*

Caroline Cheng, PhD

Erasmus MC, University Medical Center Rotterdam

[c.cheng@erasmusmc.nl](mailto:c.cheng@erasmusmc.nl)

University Medical Center Utrecht

[K.L.Cheng-2@umcutrecht.nl](mailto:K.L.Cheng-2@umcutrecht.nl)

**Supplemental tables**

**Supp. table 1. Primers**

| **Gene** | **Forward primer sequence** | **Reverse primer sequence** |  | **Product size** |  |
| --- | --- | --- | --- | --- | --- |
| CKLF1 | ccggaagcttctgttcaatc | tcatgcacaggctttttctg |  | 54bp |  |
| CMTM1 | ctccggttgtttcaagttcc | ggggaggtagccaacagc |  | 168bp |  |
| CMTM2 | cgtggtctttgctgtgagaa | gggtcctttttcctttcctg |  | 181bp |  |
| CMTM3 | accatcgtgtttgcaactga | gcagagtccccttgtttgag |  | 74bp |  |
| CMTM4 | ctggcgtcttgctgattatg | atttctgctccggctctatg |  | 160bp |  |
| CMTM5 | tctaagcaaggggagggatt | aggcacttcgagaaacctga |  | 200bp |  |
| CMTM6 | tcatatgccaacaggggaat | tgcaccttggttgttgatgt |  | 223bp |  |
| CMTM7 | acgcctcacttcaggaaaga | tgttttctgccttcctgctt |  | 208bp |  |
| CMTM8 | ttggctgggtcatgtttgta | cccagctgttgaagttgtga |  | 219bp |  |
| β-actin | tccctggagaagagctacga | agcactgtgttggcgtacag |  | 194bp |  |

**Supp. table 2. siRNA sequences**

| **Target gene** | **Target sequence** |
| --- | --- |
| Non-targeting SMARTpool | UGGUUUACAUGUCGACUAA |
|  | AGGUUUACAUGUUGUGUGA |
|  | UGGUUUACAUGUUUUCUGA |
|  | UGGUUUACAUGUUUUCCUA |
| CMTM4 siGENOME set 1 | AGAAAUUGCUGCCGUGAUA |
| CMTM4 siGENOME set 2 | ACACUGGACUCAGCGUUU |
| CMTM4 SMARTpool | CAAUCGUACUGGCUGCUUU |
|  | ACUGGCGUCUUGCUGAUUA |
|  | AGAAAUUGCUGCCGUGAUA |
|  | AAACGUAACAGUAAGGAAA |
| CMTM3 SMARTpool | GCCCUCAUCUACUUUGCUA |
|  | GCAACUGAUUUCUACCUGA |
|  | CAAGACAGAAGAAGAGAGAAU |
|  | UUAACGACGUGGCCAAAUU |
| Rab4a SMARTpool | GCUCAGGAGUGUGGUUGUU |
|  | UACAAUGCGCUUACUAAUU |
|  | GAUAAUAAAUGUUGGUGGU |
|  | GAACGAUUCAGGUCCGUGA |

**Supp. table 3. Antibodies**

| **Antibody** | **Company** |
| --- | --- |
| Rabbit pAb anti-CMTM4 | Sigma Aldrich |
| Rabbit mAb anti-CMTM4 | Santa cruz |
| Mouse mAb anti-CMTM4 | Abcam |
| Rabbit pAb anti-CMTM3 | Sigma-aldrich |
| Rabbit anti-β-actin | Cell Signaling Technology |
| Goat anti-β-actin | Abcam |
| Rhodamin phalloidin | Invitrogen |
| Rabbit mAb VE-cadherin (D87F2) XP | Cell Signaling Technology |
| Rabbit (DA1E) mAb IgG XP Isotype control | Cell Signaling Technology |
| Mouse mAb anti-Rab7 | Sigma-aldrich |
| Rabbit pAb anti-Rab7 | Sigma-aldrich |
| Mouse mAb anti-Rab4 (clone 4E11) | LsBio |
| Rabbit pAb anti-Rab4 | Abcam |
| Mouse mAb anti-Rab11 (clone 47) | BD Transduction Laboratories |
| Mouse mAb anti-clathrin Heavy Chain Clone 23 | BD Transduction Laboratories |
| Mouse mAb anti-EEA1 (early endosome antigen 1) | BD Transduction Laboratories |
| Mouse anti-VE-cadherin | Sigma-aldrich |
| Alexa Fluor 488 goat anti mouse IgG | Invitrogen |
| Alexa Fluor 488 goat anti rabbit IgG | Invitrogen |
| Alexa Fluor 568 donkey anti rabbit | Invitrogen |
| Alexa Fluor 594 goat anti rabbit IgG | Invitrogen |
| IRDye 800CW donkey anti-mouse | Li-cor Biosciences |
| IRDye 800CW goat anti-rabbit | Li-cor Biosciences |
| IRDye 800CW donkey anti-goat | Li-cor Biosciences |
| IRDye 680RD donkey anti-goat | Li-cor Biosciences |
| IRDye 680RD donkey anti-rabbit | Li-cor Biosciences |
| IRDye 680RD goat anti-mouse | Li-cor Biosciences |

**Supp. table 4. CMTM4 morpholino sequences**

| **Name** | **Antisense morpholino sequence** |
| --- | --- |
| CMTM4 i1-e2 | CCAGAACCTGAGAAGAAGGAGGAGA |
| CMTM4 i2-e3 | AAATCCTGCAAATGCGAGGAAGAGA |

**Supplemental figures**

**Supp. Fig. 1**

**
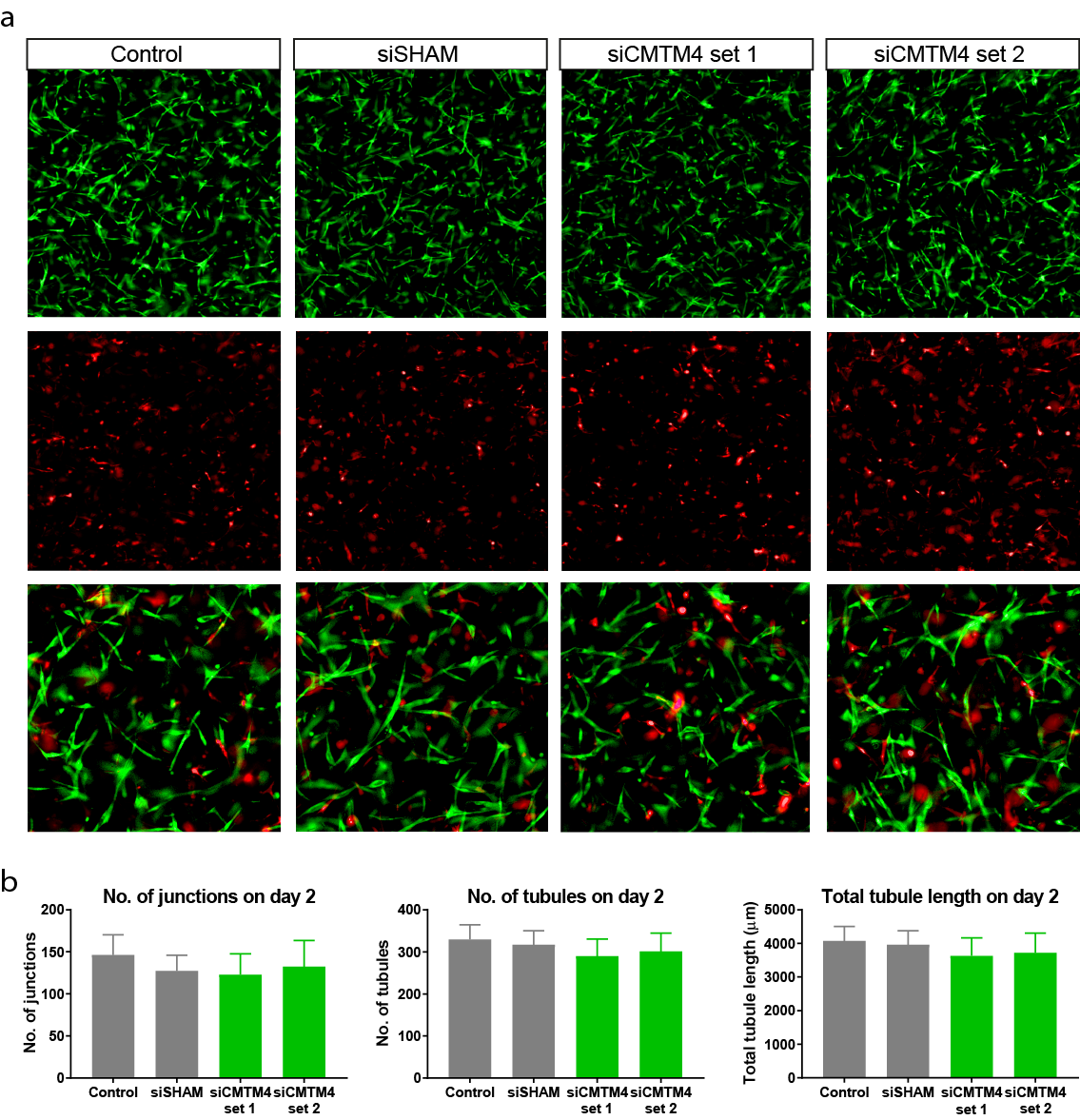
**

**
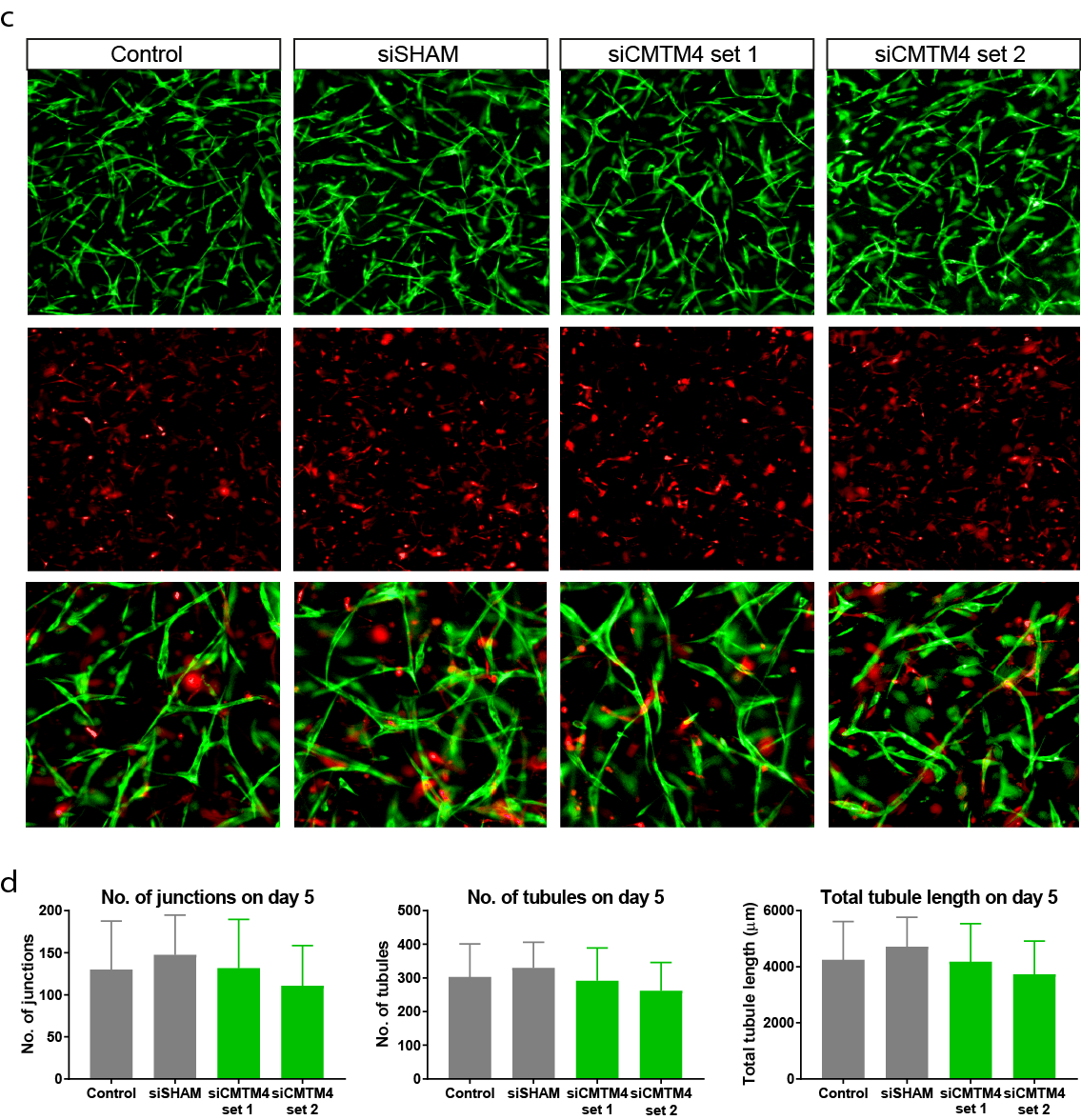
**

**Supp. Fig. 1. CMTM4 silencing in pericytes does not affect vascular growth *in* *vitro***

**a** Representative immunofluorescent images taken at 2x magnification (upper rows) and zoomed-in images (lower row) of GFP-labelled HUVECs (green) and dsRED-labelled pericytes (red) cultured for 2 days in a 3D collagen matrix, in which the dsRED labeled pericytes were transfected with either CMTM4-targeting siRNA (siCMTM4 set 1 or set 2), non-targeting siRNA (siSHAM) or not transfected (control). **b** Quantification of the number of junctions, number of tubules and total tubule length of siCMTM4 set 1 and set 2, siSHAM and control HUVECs at day 2 of co-culture. Values are mean ± SEM; N=4 co-cultures. **c** Representative immunofluorescent images taken at 2x magnification (upper rows) and zoomed-in images (lower row) of GFP-labelled HUVECs (green) and dsRED-labelled pericytes (red) cultured for 5 days in a 3D collagen matrix, in which the dsRED labeled pericytes were transfected with either CMTM4-targeting siRNA (siCMTM4 set 1 or set 2), non-targeting siRNA (siSHAM) or not transfected (control). **d** Quantification of the number of junctions, number of tubules and total tubule length of siCMTM4 set 1 and set 2, siSHAM and control HUVECs at day 5 of co-culture. Values are mean ± SEM; N=4 co-cultures.

**Supp. Fig. 2**

**
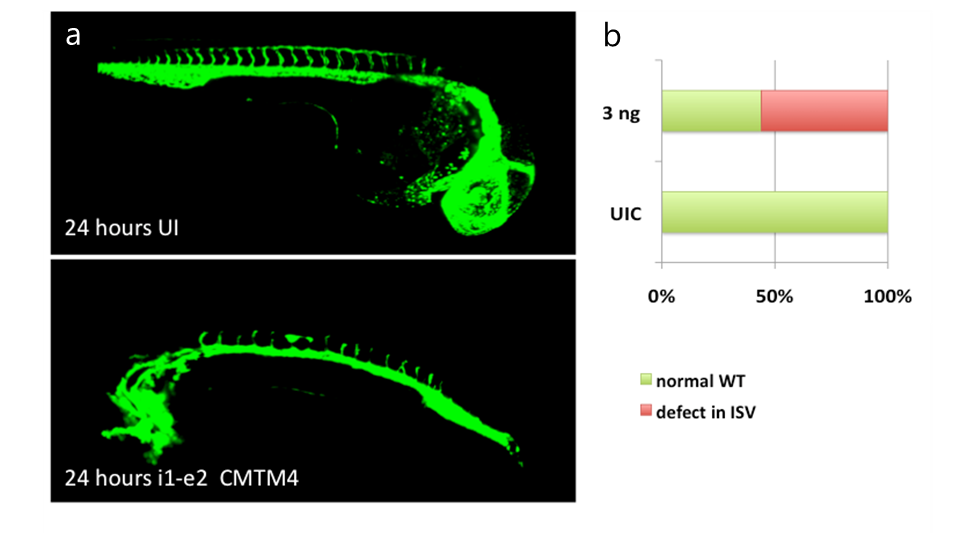
**

**Supp. Fig. 2. Silencing of CMTM4 by morpholino targeting in developing zebrafish larvae triggers defects in intersomitic vessels**

**a** Tg(fli1:eGFP)_y1_ larvae at 24 hours post fertilization. Defects in intersomitic vessel formation were detected in the trunk region in larvae injected with morpholinos targeting the splice site of i1-e2 of CMTM4 (indicated as i1-e2 CMTM4) (lower image) compared to uninjected (UI) controls (upper image). Vasculature is visualized with eGFP (green). 2x magnification **b** Quantification of intersomitic vessel defect phenotype versus wildtype phenotype in i1-e2 injected group versus UI controls (UIC). Data represents percentage of counted larvae (~100 counted per group).

**Supp. Fig. 3**


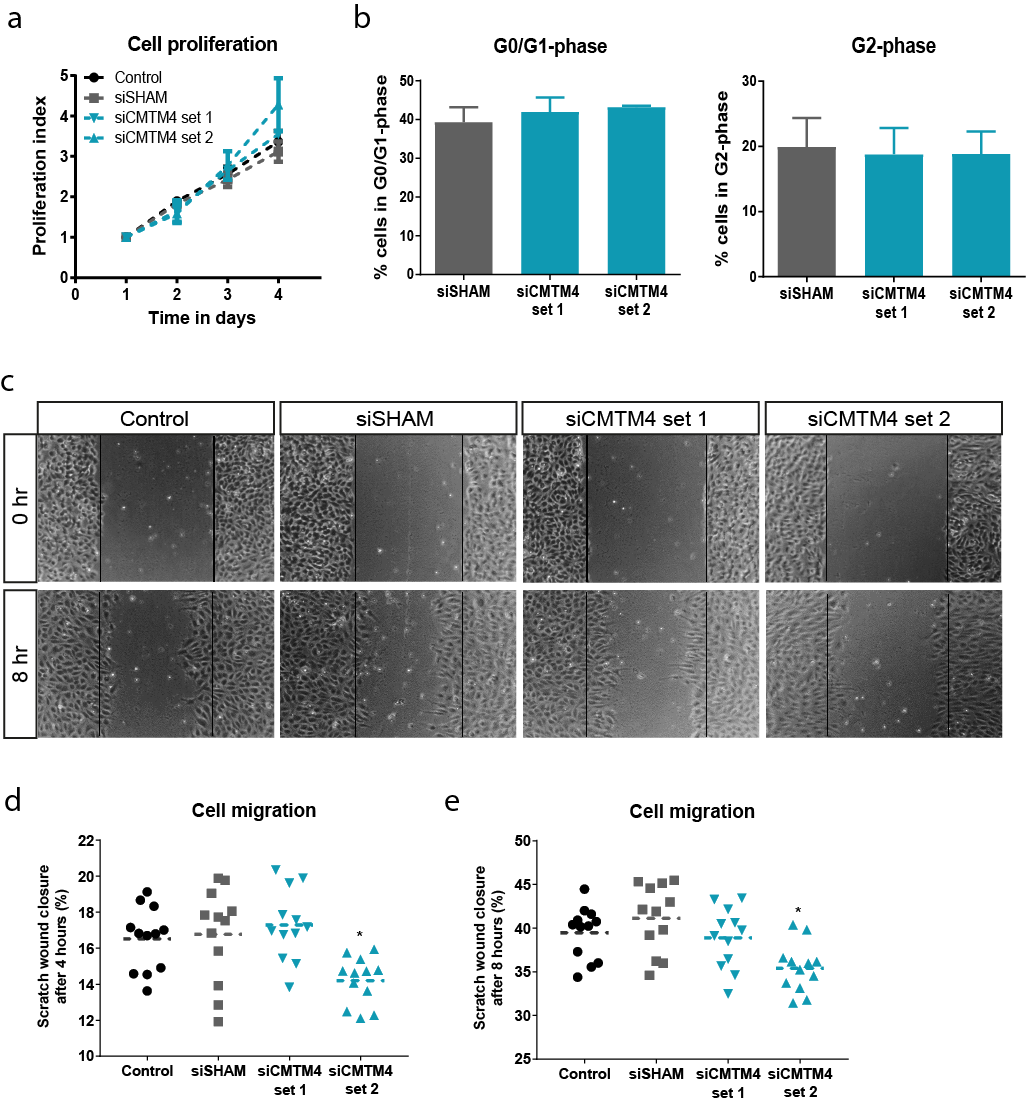


**Supp. Fig. 3. CMTM4 silencing does not affect endothelial cell proliferation, but has a small effect on cell migration**

**a** The number of cells counted at day of seeding (day 1), and at day 2, 3 and 4 of cell proliferation in siCMTM4 HUVECs (set 1 and set 2) compared with siSHAM and non-transfected controls. Shown is mean ± SEM, N=4. **b** Bargraphs showing the % of G0/G1 and G2 phase cells at day 2 after transfection in siCMTM4 versus siSHAM and non-transfected controls. Shown is mean ± SEM, N≥3. **c** Representative brightfield microscope images (4x magnification) of a scratch migration assay after 8 hours of migration of siCMTM4 HUVECs (set 1 and set 2) compared with siSHAM and non-treated controls. Bargraphs of the quantified results of the scratch migration assay showing the % area within the scratched region covered by siCMTM4 (set 1 and set 2) HUVECs compared with siSHAM and non-treated controls after (**d**) 4 hours and (**e**) 8 hours. Shown is mean; N=2, 6 wells each.

**Supp. Fig. 4**

**
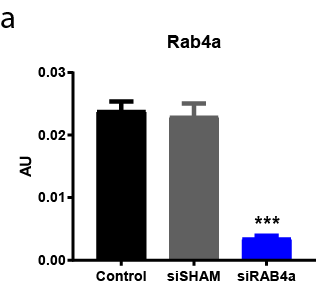
**

**Supp. Fig. 4. Rab4a-targeting siRNA induces significant silencing of Rab4a expression**

**a** Gene expression levels of Rab4a in HUVECs transfected with Rab4a-targeting siRNA (siRAB4a), non-targeting siRNA (siSHAM) and in non-transfected HUVECs (control). Gene expression levels were normalized to β-actin (AU); values are mean ± SEM; N=4 qPCRs; ***P<0.001 compared to both control and siSHAM.

**Supp. Fig. 5**

**
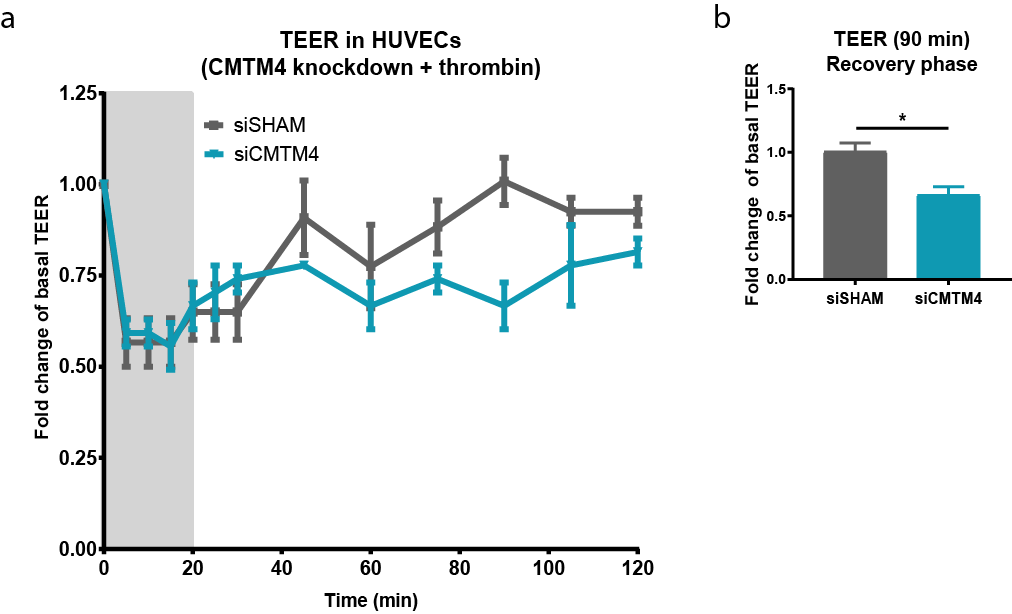
**

**Supp. Fig. 5. CMTM4 promotes restoration of endothelial electric resistance in thrombin-induced response**

**a** Thrombin response (presented in fold change compared with basal resistance of nontransfected group: Y axes) of confluent HUVEC monolayers during (0–20 minutes) and after (20–120 minutes) thrombin (1 U/mL) stimulation in siCMTM4 and siSHAM conditions. At 0 minutes, thrombin was added and at 20 minutes thrombin was removed (gray area). Mean ± SEM. N=3 per time series. **b** Increase in resistance during recovery phase at 90 minutes in siSHAM and siCMTM4 groups. Mean ± SEM. *P≤0.05 versus siSHAM. N=3.

**Supp. Fig. 6**

**
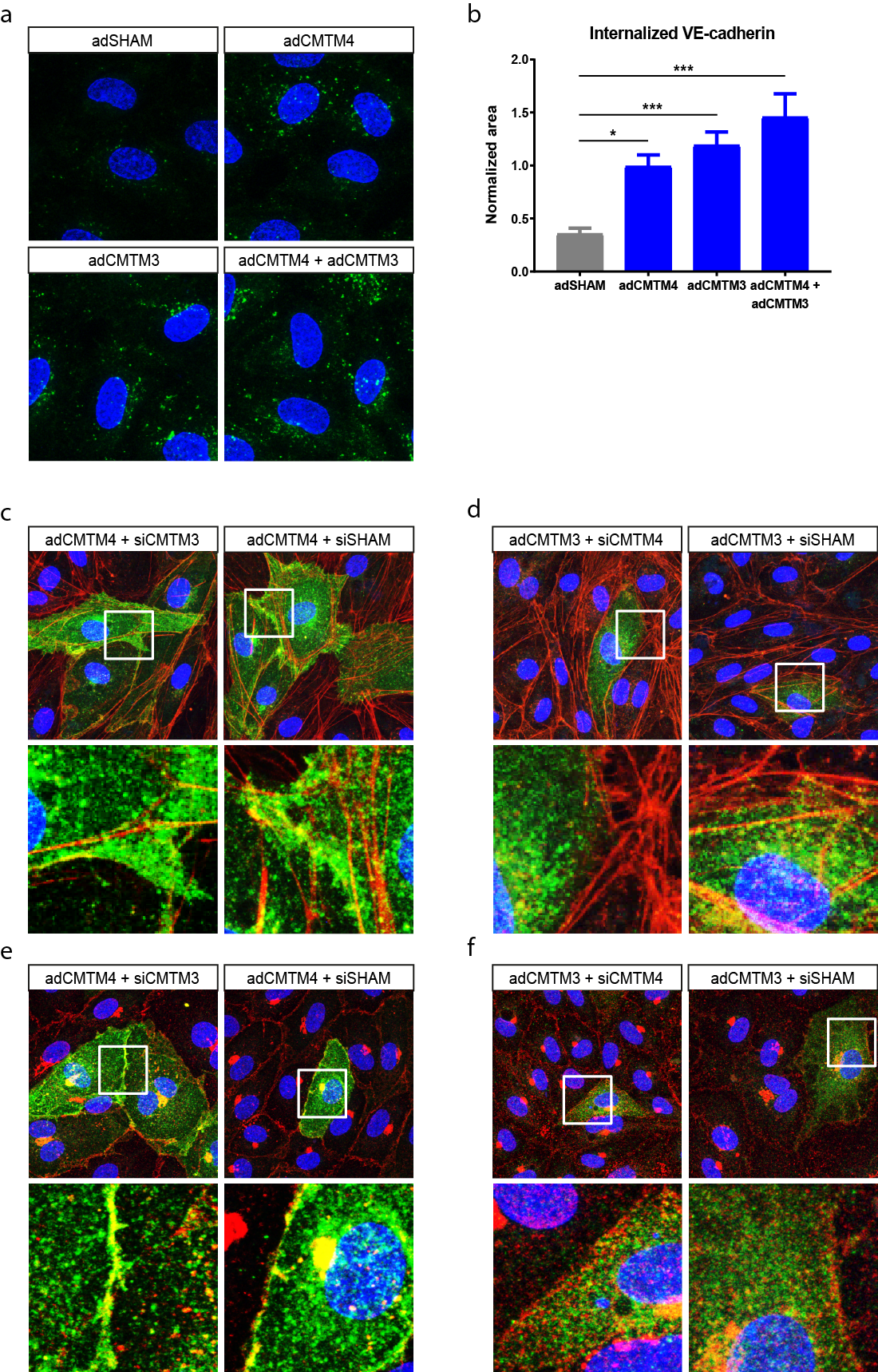
**

**
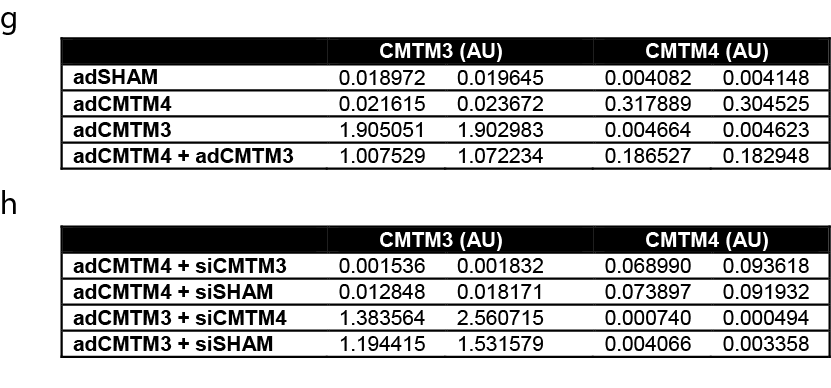
**

**Supp. Fig. 6. CMTM4 and CMTM3 do not interfere or enhance each other’s function and localization**

**a** Representative zoomed-in confocal micrographs (63x magnification) of confluent adSHAM, adCMTM4 (VPC20), adCMTM3 (VPC20) and adCMTM4 + adCMTM4 double overexpression (VPC10 each) HUVECs labeled with VE-cadherin antibodies at 4°C, followed by 37°C incubation for 1 hour to track VE-cadherin movement. Surface bound VE-cadherin antibodies were removed by an acid-wash, before proceeding with immunostaining for visualization of internalized VE-cadherin (green). **b** Quantification of internalized VE-cadherin area per cell in adSHAM, adCMTM4 (VPC20), adCMTM3 (VPC20) and adCMTM4 + adCMTM4 double overexpression (VPC10 each) HUVECs. *P<0.05, ***P0.001 versus adSHAM. N=2, 5 Z-stacks each. **c** Representative 63x magnification (upper row) and zoomed-in (lower row) confocal microscope images of adCMTM4 + siCMTM3 and adCMTM4 + siSHAM HUVECs immunostained for CMTM4 (green) and F-actin (red) (VPC10). **d** Representative 63x magnification (upper row) and zoomed-in (lower row) confocal microscope images of adCMTM3 + siCMTM4 and adCMTM3 + siSHAM HUVECs immunostained for CMTM3 (green) and F-actin (red) (VPC10). **e** Representative 63x magnification (upper row) and zoomed-in (lower row) confocal microscope images of adCMTM4 + siCMTM3 and adCMTM4 + siSHAM HUVECs immunostained for CMTM4 (green) and VE-cadherin (red) (VPC10). **f** Representative 63x magnification (upper row) and zoomed-in (lower row) confocal microscope images of adCMTM3 + siCMTM4 and adCMTM3 + siSHAM HUVECs immunostained for CMTM3 (green) and VE-cadherin (red) (VPC10). **g** Gene expression levels of CMTM3 and CMTM4 in adSHAM, adCMTM4 (VPC20), adCMTM3 (VPC20) and adCMTM4 + adCMTM4 double overexpression (VPC20) HUVECs. Gene expression levels were normalized to β-actin (AU); N=2 qPCRs. **h** Gene expression levels of CMTM3 and CMTM4 in adCMTM4 + siCMTM3, adCMTM4 + siSHAM, adCMTM3 + siCMTM4 and adCMTM3 + siSHAM HUVECs. Gene expression levels were normalized to β-actin (AU); N=2 qPCRs; VPC10.
